# The *Sinorhizobium fredii* HH103 Lipopolysaccharide Is Not Only Relevant at Early Soybean Nodulation Stages but Also for Symbiosome Stability in Mature Nodules

**DOI:** 10.1371/journal.pone.0074717

**Published:** 2013-10-01

**Authors:** Isabel Margaret, M. Mercedes Lucas, Sebastián Acosta-Jurado, Ana M. Buendía-Clavería, Elena Fedorova, Ángeles Hidalgo, Miguel A. Rodríguez-Carvajal, Dulce N. Rodriguez-Navarro, José E. Ruiz-Sainz, José M. Vinardell

**Affiliations:** 1 Departamento de Microbiología, Facultad de Biología, Universidad de Sevilla. Sevilla, Spain; 2 Instituto de Ciencias Agrarias, CSIC, Madrid, Spain; 3 Departamento de Química Orgánica, Facultad de Química, Universidad de Sevilla, Sevilla, Spain; 4 IFAPA, Centro Las Torres-Tomejil, Alcalá del Río (Sevilla), Spain; The Ohio State University, United States of America

## Abstract

In this work we have characterised the *Sinorhizobium fredii* HH103 *greA lpsB lpsCDE* genetic region and analysed for the first time the symbiotic performance of *Sinorhizobium fredii lps* mutants on soybean. The organization of the *S. fredii* HH103 *greA*, *lpsB*, and *lpsCDE* genes was equal to that of *Sinorhizobium meliloti* 1021. *S. fredii* HH103 *greA*, *lpsB*, and *lpsE* mutant derivatives produced altered LPS profiles that were characteristic of the gene mutated. In addition, *S. fredii* HH103 *greA* mutants showed a reduction in bacterial mobility and an increase of auto-agglutination in liquid cultures. RT-PCR and qPCR experiments demonstrated that the HH103 *greA* gene has a positive effect on the transcription of *lpsB*. Soybean plants inoculated with HH103 *greA*, *lpsB* or *lpsE* mutants formed numerous ineffective pseudonodules and showed severe symptoms of nitrogen starvation. However, HH103 *greA* and *lps* mutants were also able to induce the formation of a reduced number of soybean nodules of normal external morphology, allowing the possibility of studying the importance of bacterial LPS in later stages of the *S. fredii* HH103-soybean symbiosis. The infected cells of these nodules showed signs of early termination of symbiosis and lytical clearance of bacteroids. These cells also had very thick walls and accumulation of phenolic-like compounds, pointing to induced defense reactions. Our results show the importance of bacterial LPS in later stages of the *S. fredii* HH103-soybean symbiosis and their role in preventing host cell defense reactions. *S. fredii* HH103 *lpsB* mutants also showed reduced nodulation with *Vigna unguiculata*, although the symbiotic impairment was less pronounced than in soybean.

## Introduction

The lipopolysaccharide (LPS) is a complex glycolipid component present in the outer leaflet of the Gram-negative bacteria, including rhizobia [1]–[3]. The LPS of rhizobia shows the same general architecture as that from LPS of enteric and animal Gram-negative pathogens. It can be conceptually divided into three different structural regions: an inner acylated saccharide known as lipid A that is linked to a central core oligosaccharide which is attached to an outer O-chain polysaccharide [4]. A single preparation of rhizobial LPS contains a mixture of LPS molecules. This mixture is usually composed of molecules that carry various lengths of the O-chain polysaccharide, core oligosaccharide, and lipid A, but also contains molecules which are devoid of the O-chain polysaccharide [2]. LPS molecules with O-chain polysaccharide are often referred to as “smooth” LPS (or S-LPS), and those without O-chain as rough LPS, or R-LPS. LPS molecules carrying truncated (shorter) O-chains (“semi-rough” or “semi-smooth” LPS) can also be observed, particularly in some mutants [5].

LPS may play significant roles at different stages of the *(Brady/Sino/Meso)Rhizobium*-legume symbioses, such as initial recognition and infection, invasion of root cortical cells, bacterial penetration into plant cells, formation and persistence of functional symbiosomes and nodule senescence. LPS may act as inhibitors of plant defence responses, as bacterial signals, as a physical passive barrier, or as antigens mimicking a plant-like interface [2], [6], [7].

O-chain rhizobial mutants are defective in indeterminate-nodule forming legumes (such as *Pisum sativum* or *Medicago sativa*) in the endocytotic invasion of the root nodule cells [8]–[11]. The presence of an intact LPS is also necessary for the formation of effective determinate nodules. Thus, *Rhizobium etli*, *Bradyrhizobium japonicum,* and *B. elkanii* mutants that lacked the O-chain are defective in their symbioses with determinate-nodule forming legumes, such as *Phaseolus vulgaris* or *Glycine max*. In some cases, nodules are not formed while in others the small white nodules formed (pseudonodules) contain aborted infection threads, show severe structural aberrations, and are devoid of bacteria and leghaemoglobin [12]–[14]. The symbiotic phenotypes reported for rhizobial LPS mutants clearly indicate that the O-chain polysaccharide is necessary to form a normal nitrogen-fixing symbiosis, and that structural changes can mainly occur to the O-chain and to the Lipid A portion of the LPS during the transition from a vegetative bacterium to the functional bacteroid [1].

*Sinorhizobium fredii* HH103 is a fast growing rhizobial strain that nodulates *Glycine max* (soybean) and many other determinate- and indeterminate-nodule forming legumes [15]. The genome sequence of *S. fredii* HH103 has been nearly completed and is available in the EMBL Nucleotide Sequence Database (EMBL-Bank) under accession numbers HE616890 to HE616899 [15], [16]. *S. fredii* HH103 produces at least five different surface polysaccharides: exopolysaccharides (EPS), lipopolysaccharides (LPS), capsular polysaccharides (KPS [K-antigen polysaccharides]), and cyclic glucans (CG). Two different types of KPS are constitutively produced by *S. fredii* HH103. One of them, called poly-PseAc, is a homopolymer of a derivative of the pseudaminic acid [17], while the other is a homopolymer of 3-deoxy-D-*manno*-oct-2-ulosonic acid (Kdo) [18]. The chemical structure of the *S. fredii* HH103 CG has been determined [19].

*S. fredii* HH103 mutants affected in the production of KPS, CG or EPS have been already constructed and described. HH103 mutants unable to produce EPS are fully effective with soybeans [20], while those unable to produce CG only form small knot-like structures (pseudonodules) that do not fix nitrogen and are devoid of rhizobial cells [19]. The HH103 poly-PseAc (hereafter called “KPS”) plays an important role in the *S. fredii*-soybean symbiosis, since mutants affected in genes of the *rkp-1* and *rkp-3* regions are symbiotically impaired with soybean [20]–[23]. All these studies led to the conclusion that KPS and CG, but not EPS, are relevant for the capacity of *S. fredii* HH103 to nodulate soybean.

*Sinorhizobium meliloti* and *S. fredii* LPS core regions are closely related [2]. The core oligosaccharide of the *S. fredii* USDA257 LPS is composed of Kdo, glucose, galactose, glucuronic acid, and galacturonic acid [24]. This carbohydrate composition is similar to that described for the LPS core of *S. meliloti* 1021 [9]. To our knowledge, neither the structure of the S. *fredii* HH103 LPS nor the symbiotic capacity of any *lps* mutant has ever been studied.

In this study we constructed *S. fredii* HH103 mutants affected in two genes (*lpsB* and *lpsE*) coding for glycosyl transferases involved in LPS biosynthesis, and in the *greA* gene, which is located close to *lpsB* and codes for an elongation transcriptional factor. The *greA lpsB lpsCDE* cluster is well conserved in the genus *Sinorhizobium* as well as in *Bradyrhizobium japonicum* [11], [25]. Although *S. meliloti* 2011 *greA*, *lpsB*, *lpsE*, *lpsD*, and *lpsC* mutants show all altered LPS profiles, only the *greA* mutants are symbiotically impaired with *Medicago sativa*, an indeterminate nodule-forming legume [11]. In NGR234, a *S. fredii* strain that does not nodulate soybeans, mutation of the *lpsB* gene not only alters the LPS profile but also eliminates (an average of less than one nodule per plant) the bacterial nodulation capacity with *Vigna unguiculata* [25]. Up to now, the effects of *greA*, *lpsB* or *lpsE* mutations on the bacterial symbiotic capacity had not been investigated in rhizobia able to nodulate soybean. Here we show that mutations in the *S. fredii* HH103 *greA*, *lpsB*, and *lpsE* genes also provoke alterations in the LPS structure. In contrast to the situation described for the *S. meliloti*-alfalfa symbiotic interaction, all these mutations impaired the *S. fredii* HH103-soybean symbiosis. Light and transmission electron microscopy (TEM) studies of soybean nodules displaying normal external morphology clearly showed the onset of early nodule senescence.

## Results

### Isolation of the *S. fredii* HH103 *greA lpsB lpsCDE* Genes

**Figure 1:**
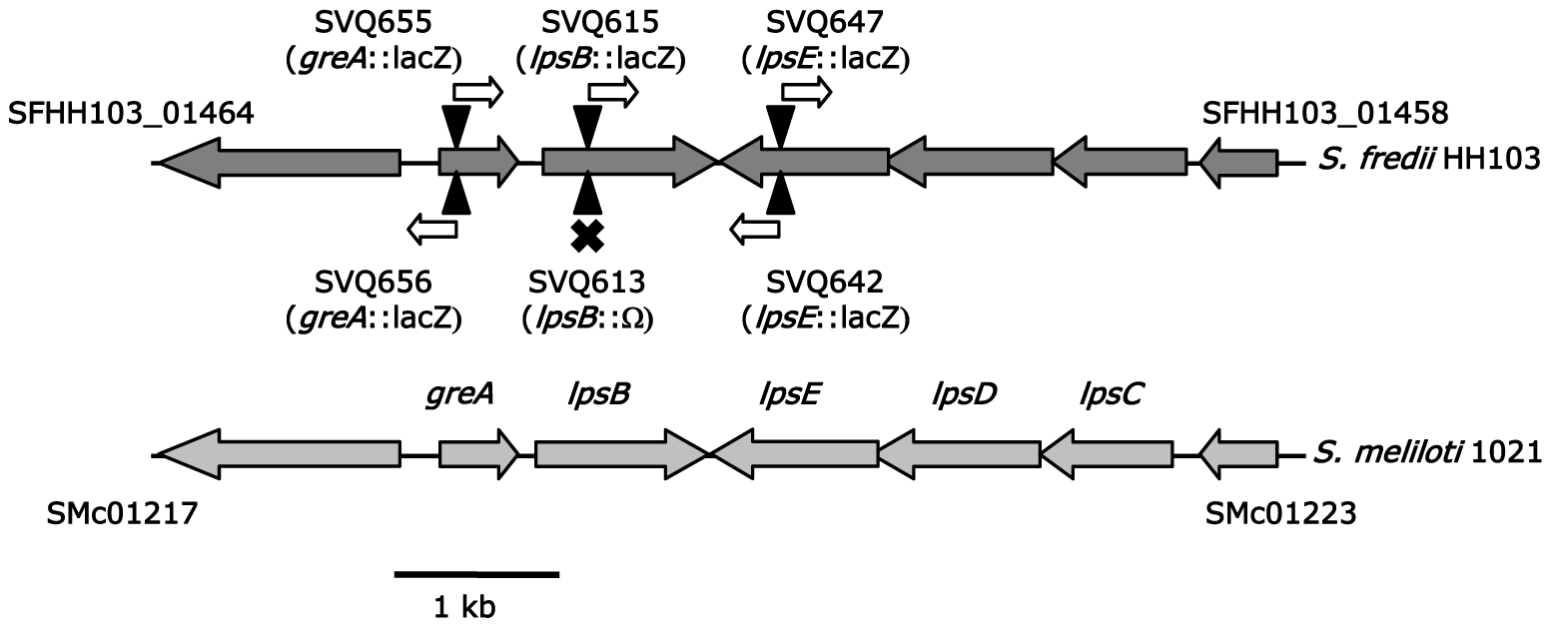
Genetic organization of the *greA-lpsB-lpsCDE* region of *S. fredii* HH103 and a comparison to that of *Sinorhizobium meliloti* 1021. Black triangles indicate the position at which the *lacZ*Δp-Gm^R^ cassette (in mutants SVQ655, SVQ656, SVQ615, and SVQ642) or the Omega interposon (in mutant SVQ613) were inserted to generate *S. fredii* HH103 mutants. The predicted effect of the cassette on transcription is showed as a white arrow (indicating direction of transcription from the *aacC1* gene belonging to *lacZ*Δp-Gm^R^) or a black cross (indicating both transcriptional and translational blocking due to Ω).

Before the HH103 genome sequence became available [16], we employed a previously described PCR-based screening of an HH103 genomic library [19] to isolate cosmid pMUS908 and sequenced a 5646-bp DNA fragment containing the HH103 *greA lpsB lpsCDE* genes (accession number JX170205). This fragment matches to nucleotides 1578624 to 1572979 of the HH103 chromosome sequence (HE616890). *In silico* analysis of the sequenced fragment revealed that the *S. fredii* HH103 *greA lpsB lpsCDE* genes show the same genetic organization that in *S. meliloti* 1021 (Figure 1). The putative proteins encoded by these ORFs have 158, 351, 270, 343, and 340 residues and are 98, 83, 82, 80, and 80% identical, respectively, to the corresponding orthologues of *S. meliloti* 1021. The *greA* gene, located upstream of *lpsB*, encodes a transcription elongation factor. The *lpsCDE* genes are transcribed in the opposite direction that *greA* and *lpsB*. The products of *lpsB lpsCDE* are glycosyl transferases putatively involved in LPS core biosynthesis.

In order to investigate the relevance of the *lpsB lpsCDE* genes of *S. fredii* HH103 in different bacterial characteristics, such as LPS synthesis and symbiotic capacity, different mutants (Figure 1 and Table 1) were generated as described in Material and Methods: SVQ613 (*lpsB*::Ω), SVQ615 (*lpsB*::*lacZ*Δp-Gm^R^, →→), SVQ642 (*lpsE*::*lacZ*Δp-Gm^R^, →→), and SVQ647 (*lpsE*::*lacZ*Δp-Gm^R^, →←). In some mutants the mutated gene and the *lacZ*Δp-Gm^R^ cassette had the same transcriptional orientation (→→), while in others was opposite (→←).We were also interested in investigating whether *greA* could have an effect on *lpsB* expression and LPS production. For this purpose, we also generated two independent mutants in the *greA* gene by using the *lacZ*Δp-Gm^R^ cassette; these two mutants differ in the orientation of the *lacZ* cassette: SVQ655 (*greA*::*lacZ*Δp-Gm^R^,→→) and SVQ656 (*greA*::*lacZ*Δp-Gm^R^,→←).

**Table 1:** Bacterial strains and plasmids used in this study.

Strain or plasmid	Derivation and relevant properties	Source or reference
***S. fredii***		
HH103-Rif^R^ ( = SVQ269)	Spontaneous Rif^R^ derivative of HH103	[51]
SVQ613	HH103-Rif^R^ *lpsB*::Ω	This work
SVQ615	HH103-Rif^R^ *lpsB:: lacZ*Δp-Gm^R^, same transcriptional direction in *lacZ* and *lpsB*	This work
SVQ642	HH103-Rif^R^ *lpsE:: lacZ*Δp-Gm^R^, same transcriptional direction in *lacZ* and *lpsE*	This work
SVQ647	HH103-Rif^R^ *lpsE:: lacZ*Δp-Gm^R^, opposite transcriptional direction in *lacZ* and *lpsE*	This work
SVQ655	HH103-Rif^R^ *greA:: lacZ*Δp-Gm^R^, Same transcriptional direction in *lacZ* and *greA*	This work
SVQ656	HH103-Rif^R^ *greA:: lacZ*Δp-Gm^R^, opposite transcriptional direction in *lacZ* and *greA*	This work
***E. coli***		
DH5α	*supE44* Δ*lacU169 hsdR17 racA1 endA1 gyr96 thi-1 relA1,* Nx^R^	Stratagene
**Plasmids**		
pAB2001	Ap^R^ vector containing the *lacZ*Δp-Gm^R^ cassette	[45]
pBluescript II SK+	Cloning and sequencing vector, Ap^R^	Stratagene
pHP45Ω	Ap^R^ vector containing the Ω interposon (Spc^R^ Str^R^)	[46]
pK18mob	Cloning vector, Km^R^	[44]
pCPP46	Cloning vector, Tc^R^	[52]
pMUS908	Cosmid pLAFR1 carrying the *greA* and *lpsBEDC* genes of *S. fredii* HH103	This work
pMUS981	pK18mob +1.5-kb *Nar*I fragment of pMUS908 containing *lpsB*	This work
pMUS982	pK18mob containing *lpsB*::*lacZ*Δp-Gm^R^→→	This work
pMUS990	pK18mob containing *lpsB*::Ω	This work
pMUS1005	pK18mob +2.5-kb *Eco*RI-*Sph*I fragment of pMUS908 containing *lpsE*	This work
pMUS1008	pK18mob containing *lpsE*::*lacZ*Δp-Gm^R^→→	This work
pMUS1020	pK18mob containing *lpsE*::*lacZ*Δp-Gm^R^→←	This work
pMUS1022	pK18mob containing *greA*::*lacZ*Δp-Gm^R^→←	This work
pMUS1023	pK18mob containing *greA*::*lacZ*Δp-Gm^R^→→	This work
pMUS1139	pCPP46 containing *lpsB*	This work
pMUS1144	pK18mob containing *lpsB*	This work

### *S. fredii* HH103 *greA, lpsB,* and *lpsE* Mutants are Affected in LPS but not in KPS, CG or EPS Production

**Figure 2:**
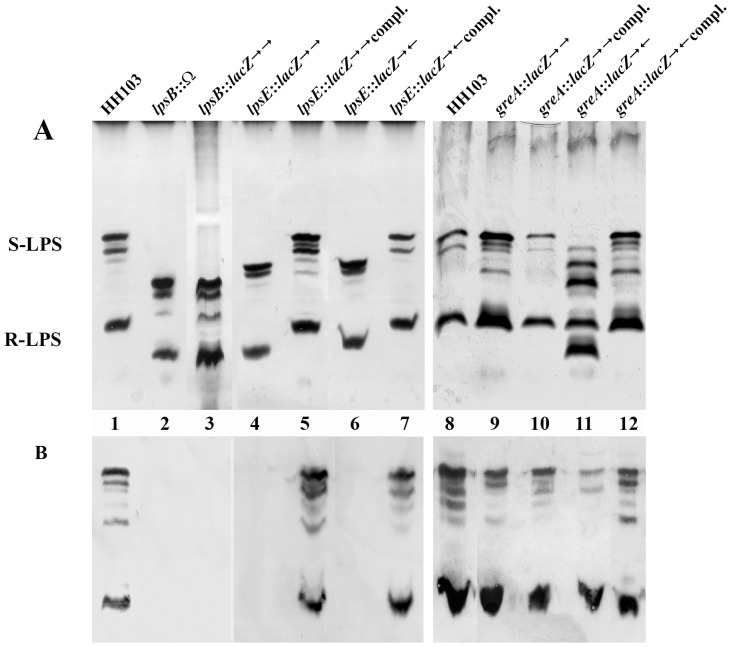
*S. fredii* HH103 *lpsB* and *lpsE* mutants and the *greA* mutant SVQ656 are affected in LPS production. **A,** sodium dodecyl sulphate-polyacrylamide gel electrophoresis and silver-staining or **B,** immuno-staining using the monoclonal antibody NB6-228.22 of lipopolysaccharides (LPS) crude extracts from *Sinorhizobium fredii* HH103 and its *lpsB* (SVQ613, SVQ615), *lpsE* (SVQ642, SVQ647), and *greA* (SVQ655, SVQ656) mutant derivatives. Lanes 1 and 8, HH103 Rif^R^; lane 2, SVQ613; lane 3, SVQ615; lane 4, SVQ642; lane 5, SVQ642 C1; lane 6, SVQ647; lane 7, SVQ647 C2; lane 9, SVQ655; lane 10, SVQ655 carrying pMUS908; lane 11, SVQ656; lane 12, SVQ656 carrying pMUS908. The rough and smooth forms of the wild-type LPS are indicated as R-LPS and S-LPS, respectively. The relevant characteristics (gene mutated, cassette employed and, when necessary, orientation of the cassette) are indicated on the top of each lane. Compl. = complemented.

The lipopolysaccharide (LPS) profiles of the *greA*, *lpsB*, and *lpsE* mutants were analyzed by PAGE experiments performed in the presence of SDS (Figure 2A) as well as by immunostaining experiments using a monoclonal antibody, NB6-228.22, that recognizes the LPS of HH103 (Figure 2B).

The S-LPS of HH103 Rif^R^ is usually composed of 5 bands [22], [23], although the number of visible bands may vary from one preparation to another (Figure 2, panel A, lanes 1 and 8). The LPS electrophoretic profiles of mutants SVQ613 (*lpsB*::Ω; lane 2), SVQ615 (*lpsB*::*lacZ*Δp-Gm^R^, →→; lane 3), SVQ642 (*lpsE*::*lacZ*Δp-Gm^R^, →→; lane 4), SVQ647 (*lpsE*::*lacZ*Δp-Gm^R^, →←; lane 6), and SVQ656 (*greA*::*lacZ*Δp-Gm^R^, →←; lanes 11) were altered in comparison with that of HH103 Rif^R^ (lanes 1 and 8). The LPS electrophoretic profile of mutant SVQ655 (*greA*::*lacZ*Δp-Gm^R^, →→; lane 9) was not apparently altered. The alteration of the profiles of *lpsB*, *lpsE,* and one of the *greA* (SVQ656) mutants consisted of an increase in the electrophoretic mobility of both S-LPS and R-LPS bands. The mobility of the S-LPS in the *lpsB* mutants (SVQ613 and SVQ615, lanes 2 and 3) was faster than that observed for mutants SVQ642, SVQ647, and SVQ656 (lanes 4, 6, and 11, respectively).

The monoclonal antibody NB6-228.22 recognized the same pattern of LPS bands in *S. fredii* HH103 Rif^R^ (Figure 2, panel B, lanes 1 and 8) and in its *greA* mutant derivative SVQ655 (lane 9). However, this antibody only recognized a subset of the S-LPS and R-LPS silver-stained bands of the *greA* mutant derivative SVQ656 (panel B, lane 11), and failed in the recognition of the fastest silver-stained bands that respectively appear in both SVQ656 LPS regions (compare lane 11 of panels A and B) These results indicate that both *greA* mutants produce LPS immunorelated with those of HH103 but SVQ656 also produces prominent new LPS bands without immunoreactivity. In addition, NB6-228.22 failed in the recognition of the altered LPS produced by mutants SVQ613 (*lpsB*::Ω, lane 2), SVQ615 (*lpsB::lacZ*Δp-Gm^R^, lane 3), and SVQ642 and SVQ647 (*lpsE::lacZ*Δp-Gm^R^, lanes 4 and 6).

Introduction of cosmid pMUS908, which contains the *greA lpsB lpsCDE* genes, into *S. fredii greA* mutants restored the wild type LPS electrophoretic and NB6-228.22 recognition patterns (Figure 2A, B). This approach failed to complement both *lpsE* and *lpsB* mutants. *S. fredii lpsE* mutants could be complemented by the introduction *in cis* of a copy of the wild type *lpsE* gene (see Material and Methods), leading to the construction of strains SVQ642C1 and SVQ647C2. All our attempts to complement the *lpsB* mutations were unsuccessful.

In *S. fredii* HH103 there are several examples of mutations that simultaneously affect the production of various surface polysaccharides [19], [21], [23]. For this reason, we have analysed the production of other surface polysaccharides in the HH103 *lps* and *greA* mutants. PAGE and/or NMR studies showed that K-antigen polysaccharides (KPS) and cyclic glucans (CG) produced by SVQ613, SVQ642, SVQ655, and SVQ656 appear to be identical to that produced by the parental strain HH103 Rif^R^ (Figures S1 and S2). All the *greA, lpsB,* and *lpsE* mutants formed colonies in TY and YMA media that were equally mucous to those formed by HH103 Rif^R^ (data not shown), suggesting that EPS production was not altered in these mutants.

### The *S. fredii* HH103 *greA* Mutants Show Reduced Mobility and their Auto-agglutination Capacity can also be Altered

**Figure 3:**
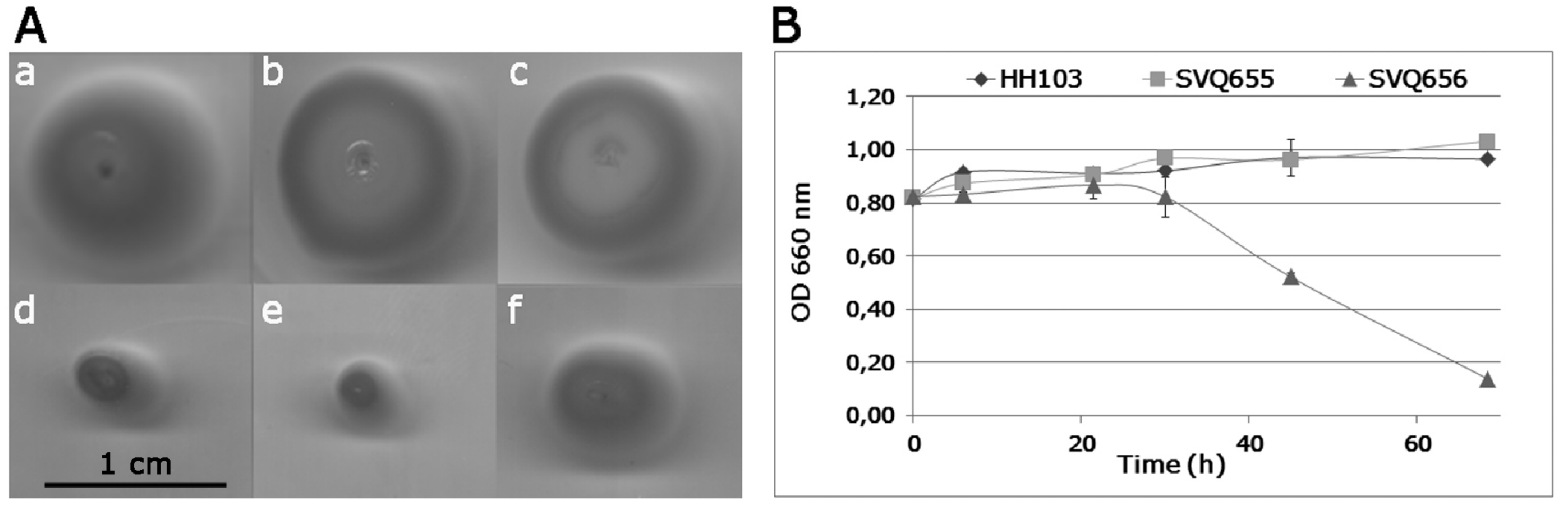
Inactivation of the *S. fredii* HH103 *greA* gene affects swimming capacity and auto-agglutination. **A**, swimming capacity of (a) *S. fredii* HH103 Rif^R^ and its (b) *lpsB* (SVQ613), (c) *lpsE* (SVQ642), (d,e) *greA* (SVQ655 and SVQ656) mutant derivatives, and (f) SVQ656 carrying cosmid pMUS908. **B**, auto-agglutination assays of *S. fredii* HH103 Rif^R^ and its *greA* (SVQ655 and SVQ656) mutant derivatives. The optical density of the cultures was monitored at 0, 6, 22, 30, 45, and 69 h of incubation at static conditions.

Surface polysaccharide alterations can affect some bacterial traits such as motility, auto-agglutination in liquid medium and biofilm formation [26], [27]. Because of this, *S. fredii* HH103 mutants affected in *greA*, *lpsB*, and *lpsE* were investigated for these capacities. Inactivation of *lpsB* or *lpsE* did not affect any of these traits (Figure 3A, Figure S3, and data not shown). Interestingly, inactivation of *greA* did not affect biofilm formation capacity either (Figure S3), but significantly diminished bacterial motility in soft (0.24% agar) TY medium (Figure 3A). In addition, the intensity of auto-agglutination of SVQ656 (*greA*::*lacZ*Δp-Gm^R^, →←) was clearly higher than that shown by HH103 Rif^R^, while that of SVQ655 (*greA*::*lacZ*Δp-Gm^R^, →→) was not apparently affected (Figure 3, panel B). Introduction of cosmid pMUS908 into HH103 *greA* mutants increased motility and diminished auto-agglutination up to wild type levels (Figure 3A and data not shown).

Mutants producing altered LPS forms are frequently more sensitive to detergents [28]. *S. fredii* HH103 and its mutant derivatives *lpsB* (SVQ613), *lpsE* (SVQ642) and *greA* (SVQ655 and SVQ656) were tested for their sensitivity to different chemical compounds that cause oxidative stress (H_2_O_2_ and paraquat) and/or are present in soybean roots, such as vanillic, ferulic, and salicylic acids and the isoflavone genistein [29]. The detergent SDS and ethanol were also included in these assays. *S. fredii* HH103 Rif^R^ and its mutant derivatives were not significantly inhibited by the presence in the disks of 5 µl of the following stock solutions: 1 M paraquat, 100 mM salicylic acid, 100 mM ferulic acid, 3.7 mM genistein, 100% ethanol, 6.9 mM SDS or 50 mM H_2_O_2_. Only mutant SVQ613 (*lpsB*) was inhibited by 100 mM vanillic acid (inhibition halo of about 5 mm around the paper disk).

### *S. fredii* HH103 *lpsB* Expression is not Affected by pH or the Presence of Flavonoids

It is known that various culture conditions, such as the presence of flavonoids or the medium pH, can affect the expression of genes involved in surface polysaccharide production [19], [25], [30]. Strain SVQ615 (*lpsB*::*lacZ*Δp-Gm^R^) was used for investigating the possible effect of different culture conditions on the expression of the *lpsB* gene. One of the conditions tested was the presence of genistein, a soybean secreted flavonoid able to induce the transcription of *S. fredii* HH103 *nod* genes [31]. β-galactosidase assays of SVQ615 cultures grown in the presence and absence of genistein showed similar LacZ activities (298.8±12.1 and 326.2±5.3 Miller units respectively). Mutant SVQ615 was also cultured in acidic- (pH 6.0), neutral- (pH7.0), and alkaline-buffered (pH 8.0) liquid YM (YMB) media. Non-buffered YMB media, with a pH value of 7.0 at the time of inoculation, was also included. β-galactosidase activity of SVQ615 cultures was similar in all the conditions tested (data not shown).

### The *S. fredii* HH103 *greA* and *lpsB* Genes are not Cotranscribed but Mutations in *greA* Reduce the Transcription of *lpsB*

**Figure 4:**
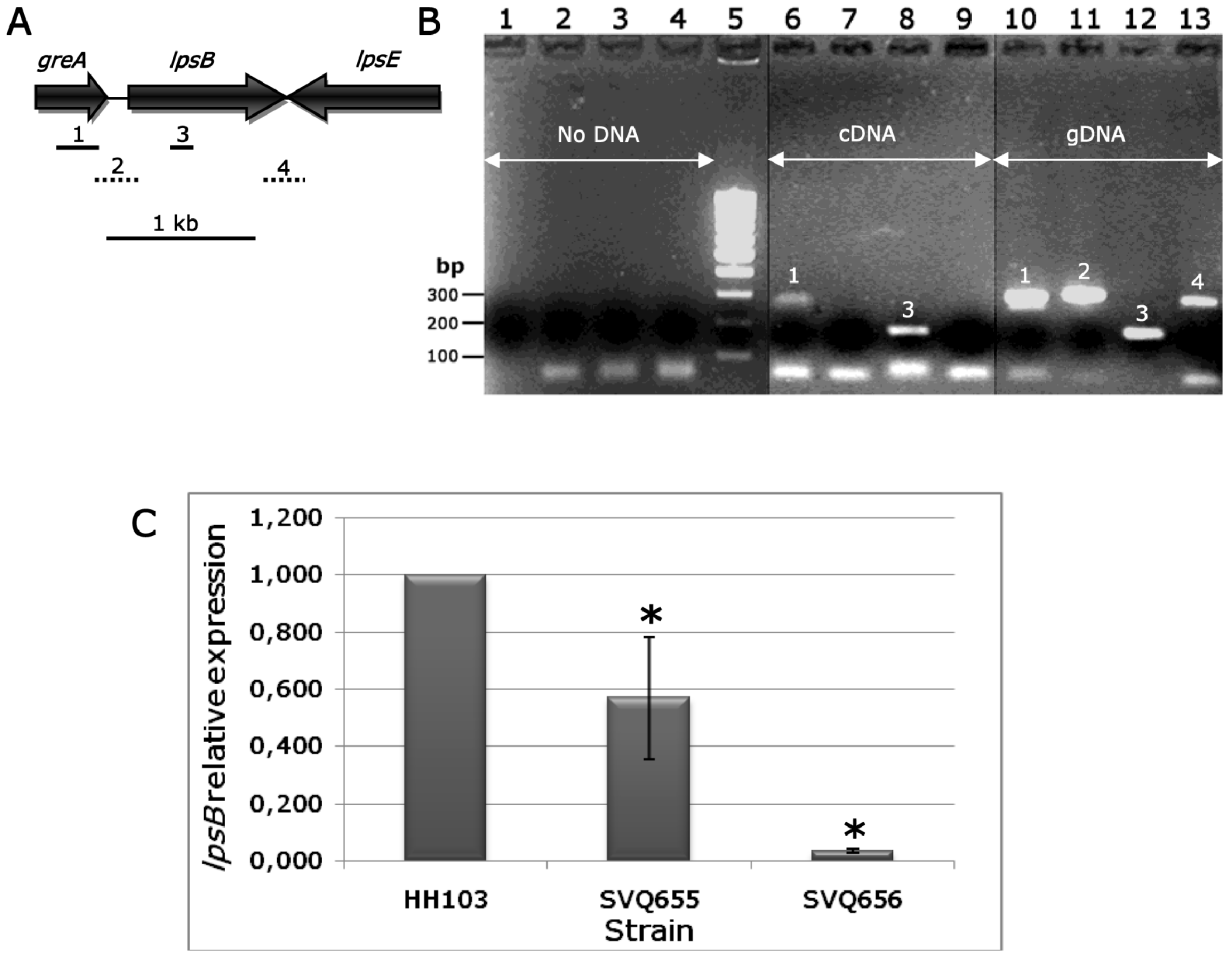
The *Sinorhizobium fredii greA* and *lpsB* genes are not cotranscribed but *greA* affects *lpsB* expression. **A,** genetic organization of the *greA-lpsB-lpsE* region. Solid lines show the position of the *greA* (1, 284-bp) and *lpsB* (3, 156-bp) internal fragments that were successfully amplified (positive controls) using cDNA or gDNA as template. Dotted lines show the positions of the PCR fragments covering the intergenic *greA*-*lpsB* (2, 156-bp) and *lpsB*-*lpsE* (4, 282-bp) regions that were amplified from gDNA but not from cDNA. **B,** agarose gel electrophoresis of samples resulting from PCR and RT-PCR experiments. lanes 1–4, controls without DNA; lane 5, 100bp-ladder DNA marker; lanes 6–9, HH103 cDNA; lanes 10–13, HH103 gDNA. Primers used: *greA*intF and *greA*intR (lanes 1, 6, and 10), *greAlpsB*-F and *greAlpsB*-R (lanes 2, 7, and 11), rt*lpsB*-F and rt*lpsB*-R (lanes 3, 8, and 1**2**), *lpsBE*-F and *lpsBE*-R (lanes 4, 9, and 13). DNA marker 100, 200, and 300-bp bands are indicated on the left of the figure. **C,** relative *lpsB* expression of *S. fredii* HH103 strains SVQ655 (*greA*::*lacZ*Δp-Gm^R^, →→) and SVQ656 (*greA*::*lacZ*Δp-Gm^R^, →←) in comparison to that of wild type HH103 Rif^R^. RNA was extracted from early exponential phase cultures grown in YMB. Each strain was pairwise compared with HH103 Rif^R^ by using the Mann-Whitney non-parametrical test. In both cases, the relative expression of *lpsB* was significantly different from that of HH103 Rif^R^ at the levels α = 5% (indicated with a black asterisk),

The *S. fredii* HH103 *greA* and *lpsB* genes are adjacent and transcribed in the same orientation. The intergenic *greA*-*lpsB* region is composed of 134 bp. The fact that the LPS electrophoretic profile of SVQ656 (in which *lacZ* and *greA* are oppositely transcribed) showed clear alterations suggests that *greA* could influence the transcription of *lpsB*. To investigate whether *greA* and *lpsB* are cotranscribed, we used RT-PCR for searching putative HH103 mRNAs covering the 3′-end of *greA* and the 5′-end of *lpsB* by using primers *greAlpsB*-F and *greAlpsB*-R (Figure 4, panels A and B; Table S1). As a positive control, primer pairs *greA*int-F/*greA*int-R and rt*lpsB*-F/rt*lpsB*-R, which allow the amplification of internal fragments of the *greA* and *lpsB* genes respectively, were employed. In addition, we used primers *lpsBE*-F/*lpsBE*-R as a negative control because they would lead to the amplification of a DNA fragment covering the 3′-end of *lpsB* and the 3′-end of *lpsE* that cannot be originated by retrotranscription of the total mRNA population since *lpsB* and *lpsE* are transcribed in opposite directions. All these primer pairs allowed the amplification of fragments of the expected size when HH103 gDNA was used as template (Figure 4, panel B, lanes 10 to 13). However, when HH103 cDNA was employed, only the internal fragments of *greA* (lane 6) and *lpsB* (lane 8) were amplified. All these results indicate that, in *S. fredii* HH103, *greA* and *lpsB* do not belong to the same transcriptional unit.

Although the *S. fredii* HH103 *greA* and *lpsB* genes are independently transcribed, the fact that the LPS of mutant SVQ656 showed an altered electrophoretic profile prompted us to investigate whether the transcription of *lpsB* was affected in a *greA*-mutant background (Figure 4, panel C). qPCR experiments showed that *lpsB* expression is about 30-fold reduced in mutant SVQ656 (0.033±0.007) when compared to HH103 (assigned the value of 1). The fact that mutant SVQ655 only presents a slight reduction of *lpsB* expression (0.569±0.215) could be due to a polar effect of the *lacZ*Δp-Gm^R^ cassette, which, in this mutant strain, has the same transcriptional orientation that *lpsB*.

### Symbiotic Properties of *S. fredii* HH103 *greA* (SVQ656), *lpsB* (SVQ613), and *lpsE* (SVQ642) Mutants

The symbiotic phenotype of *S. fredii* HH103 *greA* (SVQ655 and SVQ656), *lpsB* (SVQ613 and SVQ615), and *lpsE* (SVQ642 and SVQ647) mutants was investigated with *G. max* cv. Williams (Table 2; Figure S4). Soybean plants inoculated with any of these mutants showed severe symptoms of nitrogen starvation. All the parameters analyzed (number and fresh-weight of nodules and plant-top dry-weight) were significantly decreased in soybean plants inoculated with the *greA*, *lpsB* or *lpsE* mutants in comparison to those of plants inoculated with the wild-type parental strain HH103 Rif^R^ (Table 2). Nitrogen-fixation activity (assessed by acetylene reduction assay [ARA]) was detected in nodules showing normal external morphology (data not shown). In addition to a reduced number of Fix^+^ nodules, the *greA*, *lpsB* or *lpsE* mutants also induced the formation of pseudonodules on soybean roots. The antibiotic-resistance markers of nodule isolates were equal to those of the respective strain used as inoculant. Hence, soybean nodules formed by the different mutants tested were not due to reversions caused by the loss of the cassette or interposon used. Introduction of wild type copies of the mutated gene into HH103 *greA* or *lpsE* mutants restored wild type ability to nodulate soybean (Table 2).

**Table 2:** Plant responses to inoculation of *Glycine max* cv. Williams and *Vigna unguiculata* cv. Bisbee Red with *Sinorhizobium fredii* HH103 Rif^R^ and its *greA* or *lps* derivatives^a^.

Legume tested, inoculant^b^	Number of nodules	Nodule fresh weight (g)	Plant-top dry weight (g)
***Glycine max***			
HH103 Rif^R^	146.0±75.7	1.90±0.68	3.7±1.4
SVQ613	4.1±2.7**	0.08±0.05**	0.9±0.3**
SVQ615	3.9±3.3**	0.08±0.08**	0.6±0.2**
SVQ642	24.6±13.5**	0.37±0.21**	0.9±0.3**
SVQ642 C1	117.8±45.0	1.51±0.52	3.1±1.2
SVQ647	20.6±8.5**	0.35±0.11**	1.0±0.2**
SVQ647 C2	103.2±35.6	1.41±0.37	2.9±0.8
SVQ655	32.2±14.2**	0.43±0.17**	1.0±0.5**
SVQ655 (pMUS908)	70.2±45.7	0.75±0.45*	1.3±0.8**
SVQ656	16.9±12.7**	0.24±0.20**	0.8±0.3**
SVQ656 (pMUS908)	163.3±56.7	1.84±0.41	2.9±1.3
Uninoculated	–	–	0.9±0.5**
***Vigna unguiculata***			
HH103 Rif^R^	73.1±13.3	1.04±0.34	2.0±0.9
SVQ613	45.8±15.2*	0.80±0.43*	1.2±0.6*
Uninoculated	–	–	0.2±0.1

a Data represent averages of 8 soybean or 10 cowpea plants. Inoculated soybean and cowpea plants were grown for 55 and 43 days, respectively. Plants were grown in Leonard jars, each containing two soybean or cowpea plants. Soybean plants inoculated with *lps* and *greA* mutants also formed a high number of pseudonodules. Bacteria isolated from soybean and cowpea nodules of normal external morphology induced by each inoculant showed the resistance markers associated to the mutation (Spc^R^ or Gm^R^) or to the cosmid (Tc^R^). In SVQ655 (pMUS908), however, only 50% of nodule isolates were Tc^R^. Attempts to isolate bacteria from pseudonodules were unsuccessful.

b Each mutant was pairwise compared with the parental strain HH103 Rif^R^ by using the Mann-Whitney non-parametrical test. Numbers on the same column followed by a single or a double asterisk are significantly different from those of HH103 Rif^R^ at the levels α = 5 and 1%, respectively.

The symbiotic capacity of mutant SVQ613 (*lpsB*::Ω) was also tested with *Vigna unguiculata* cv. Bisbee Red (cowpea). Although SVQ613 was still able to induce the formation of nitrogen-fixing nodules on cowpea roots, the number of nodules formed was significantly reduced (α = 5%) when compared with the parental strain HH103 Rif^R^ (Table 2).

### *S. fredii* HH103 *greA* (SVQ656), *lpsB* (SVQ613), and *lpsE* (SVQ642) Symbiosomes Show Early Senescence in Soybean Nodules

**Figure 5:**
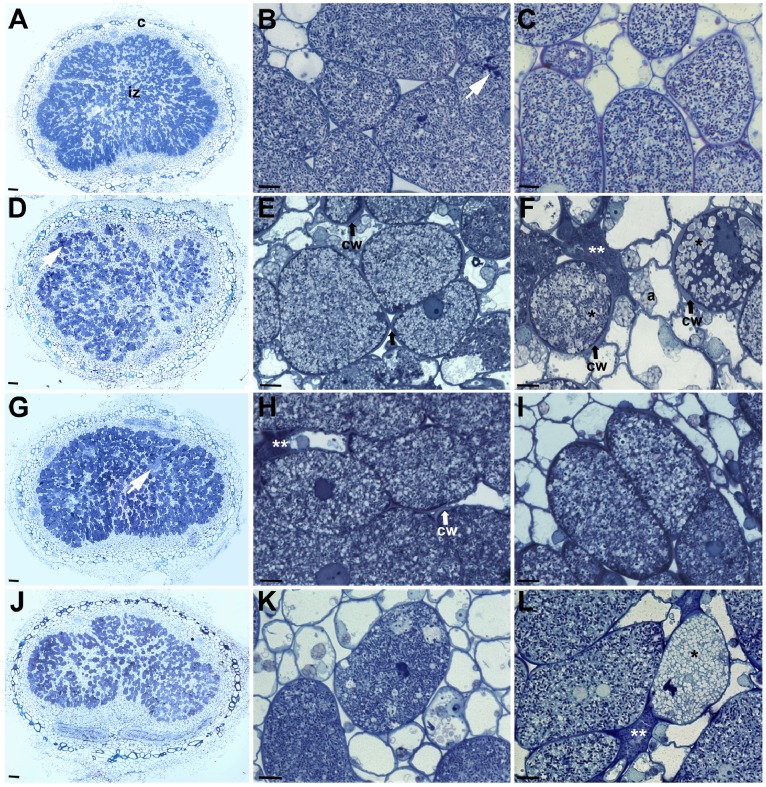
Structure of soybean nodules elicited by *Sinorhizobium fredii* strains HH103 Rif^R^ (A–C), SVQ656 (*greA*) (D–F), SVQ613 (*lpsB*) (G–I), and SVQ642 (*lpsE*) (J–L). Nodules were collected from 35^R^, showing a structured cortex and infected zone with infected and interstitial cells. B, detail of A; infected cells containing infection threads (white arrow) and symbiosomes. C, cells from an older zone than that shown in B. D, image of a SVQ656-induced nodule with evident cytological alterations (white arrow). E, F, images showing different grade of cell damage. The infected cells contain altered symbiosomes with single bacteroids and numerous vesicles (*). Completely collapsed infected cells are visible (**). Note the cell wall thickness of the infected cells. Interstitial cells show amyloplast with huge starch granules. G, image showing the structural features of an SVQ613-induced nodule. H, I, detail of cells showing similar symbiosome damages to those described above. J, structure of a SVQ642-induced nodule. K, infected cells showing different grade of structural damage. L; cell with numerous transparent vesicles (*) after lysis of bacteroids. Additional lettering and symbols: a, amyloplast; c, cortex zone; cw, cell wall; iz, infection zone. Bar size: A, D, G, J: 100 µm; B,C,E,F,H,I,K,L: 20 µm.

To understand the role of *greA*, *lpsB*, and *lpsE* in nodule development in soybean, nodules elicited by HH103 Rif^R^, SVQ656 (g*reA*), SVQ613 (*lpsB*), and SVQ642 (*lpsE*) were examined by light microscopy and transmission electron microscopy (TEM). All the mutants tested induced the formation of pseudonodules and nodules on *Glycine max* cv. Williams roots. Contrary to the peripheral vascular bundles found in normal soybean nodules (Figure 5), pseudonodules displayed the vascular tissues in a central zone, which was surrounded by sclereid and some other cells (Figure S5). No bacteria were observed by microscopy or were recovered from these pseudonodules.

**Figure 6:**
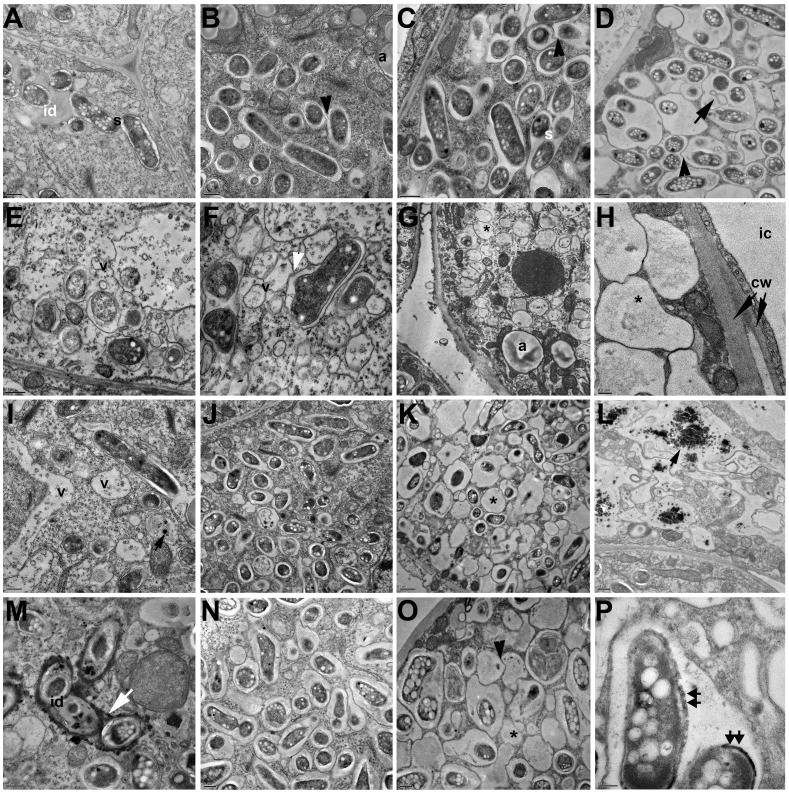
Ultrastructure characteristics of infected cells in soybean nodules elicited by *Sinorhizobium fredii* strains HH103 Rif^R^ (A–D), SVQ656 (*greA*) (E–H), SVQ613 (*lpsB*) (I–L), and SVQ642 (*lpsE*) (M–P). A, in HH103-elicited nodules, bacteria released from an infection drop (id) are surrounded by the peribacteroid membrane and forms symbiosomes (s). B, symbiosomes harboring several bacteroids after division and fusion of symbiosomes (black arrowhead). C, mature infected cell filled with symbiosomes harboring 1 to 4 bacteroids. D, older infected cell than in (C); symbiosomes display huge peribacteroid space, symbiosome fusion and ghost membranes (black arrow) can be seen. E, in SVQ656-induced nodules, freshly infected cells show numerous vesicles (v) and loss of cytoplasmic content. F, an older infected cell than (E) with an altered symbiosome, which fuses with vesicles (white arrowhead). G, image showing empty symbiosomes (*) in a cell with numerous amyloplasts. H, different cell wall thickness of an interstitial cell and of a deleterious infected cell with empty symbiosomes. I, young infected cell showing bacteroid division and vacuolar structures with osmiophilic content (black arrowhead). J, K, cells filled with single bacteroid symbiosomes. Note the presence of degenerated bacteroids and empty symbiosomes (*). L, infected cell with abundant vesicle structures containing electrodense material. M, infection drop showing osmiophilic material (white arrow). N, O, two cells with different grade of degeneration. Fusion of symbiosomes, degenerated bacteroids (black arrowhead), and empty symbiosomes (*) are shown. P, deposits of dark material on the cell wall of bacteroids (double arrows). Additional lettering and symbols: a, amyloplast; cw, cell wall; ic, interstitial cell. Bar size: A, B, C, D, E, G, I, L, M, N, O: 0.5 µm; F, H, P: 0.2 µm; J, K: 1 µm.

Since nodulation of *G. max* roots is not a synchronous process, the nodules formed vary in age and size. Therefore, the structure of nodules of different size formed on soybean roots at 21 and 35 days post inoculation (dpi) was studied (Figures 5 and 6; Figure S6). As expected, small nodules elicited by HH103 Rif^R^ displayed a cortex surrounding the central zone. This cortex consisted of an outer cortex and of an inner cortex separated by sclereid cells (Figure S6, panel A). Meristematic cells, recently infected cells, and more mature infected cells were present in the nodule central zone (panel C). Small nodules induced by the *greA* (panels B and D), *lpsB*, and *lpsE* mutants (data not shown) displayed all the nodule zones described above and the infection threads also appeared to be normal. HH103 and SVQ656 symbiosomes did not show any significant difference (panels E and F).

Light microscopy of nodules of larger size occupied by any of the inoculants tested (HH103 Rif^R^, SVQ656, SVQ613 or SVQ642) showed the typical structure of a fully grown soybean nodule (Figure 5A, D, G, J). In contrast to the apparent similarity of all the 21-dpi nodules investigated, the infected cells of 35-dpi nodules occupied by the different mutants showed cytological aberrations. The symbiosomes occupied by HH103 Rif^R^ contained several bacteroids (Fig. 5B, C), while those occupied by the mutants contained only one bacteroid, the peribacteroid space was larger, and numerous vesicles were present in the cytoplasm of host cell (Fig. 5E, F, H, I, K, L).

TEM analysis of 21-dpi nodules showed structural differences between nodules induced by the wild type and those occupied by its mutant derivatives. Following the normal symbiosome developmental program, HH103 Rif^R^ cells released from the infection droplets (Figure 6A) formed symbiosomes that were able to divide and fuse with other symbiosomes (Figure 6B). Mature symbiosomes contained 2–4 HH103 Rif^R^ bacteroids (Figure 6C). The fusion of symbiosomes in multibacteroid-containing structures and the presence of ghost membranes, which are indications of nodule senescence, were only observed in old cells (Figure 6D).

In contrast, nodules induced by the *greA*, *lpsB*, and *lpsE* mutants displayed characteristics pointing to premature termination of symbiosis (Figure 6E–J, 6N, 6P). The cytoplasm of young and mature infected cells showed a large number of endosome-like vesicles fusing with symbiosomes. Bacteroids started to be lysed inside the peribacteroid membranes. The presence of normal multi-bacteroid symbiosomes was rare. Instead, most of the symbiosomes formed by the mutants only contained a single bacteroid. Only a few fusing symbiosomes were found in some infected cells (Figure 6O). Signals of symbiosis termination by lytical clearance of bacteria, a process which is structurally similar to phagocytic elimination, were already detected in 21 dpi soybean nodules. Electron-transparent single membrane vesicles, probably provacuoles, were fusing with symbiosomes. Electro-dense and irregularly shaped bacteroids were found in vacuole-like structures, as a result of the fusion of symbiosomes with these structures (Figure 6F).

The infected cells of nodules occupied by the *greA* and *lps* mutants showed cell walls that were thicker than those of nodules induce by *S. fredii* HH103 Rif^R^ (Figure 5). This fact was especially evident in 35-dpi nodules elicited by the *greA* mutant (SVQ656). Apparently, the cell wall of interstitial cells was not affected (Figure 6H). In addition, vacuoles of cells occupied by the *lpsB* mutant (SVQ613) showed accumulations of an osmiofilic material, which might correspond to phenolic compounds (Figure 6I). Cells occupied by the *lpsE* mutant (SVQ642) contained an electron-dense material that was deposited over the membrane surrounding the unwalled infection droplet and its lumen (Figure 6M), and also between the membrane and the cell wall of bacteroids (Figure 6P).

## Discussion

Rhizobial surface polysaccharides are known to play important roles in symbiosis with legumes [7], [32]. Our group have previously characterized *S. fredii* HH103 mutants unable to produce cyclic glucans (CG) and/or capsular polysaccharides (KPS) and investigated their symbiotic properties with *Glycine max* and other legumes as well as different bacterial traits in free living conditions [19]–[23]. In this work, we have investigated the physiological and symbiotic characteristics of *S. fredii* mutants affected in genes that are directly or indirectly involved in LPS synthesis. For this purpose, different mutants in the *greA*, *lpsB*, and *lpsE* genes were constructed and analysed.

The *S. fredii* HH103 *greA*-*lpsB*-*lpsCDE* region shows the same genetic organization than that of *S. meliloti* 1021 [9], [11] or *S. fredii* NGR234 [25]. The *S. fredii* HH103 GreA protein, a transcription elongation factor, is nearly identical to those of *S. meliloti* 1021 (98% of identity) and *S. fredii* NGR234 (99% identity). The LpsB, LpsC, LpsD, and LpsE proteins are members of the type 1 glycosyltransferase family, a large family of proteins that transfer activated sugars to a variety of substrates, including glycogen and lipopolysaccharides [11], [33]. The *S. fredii* HH103 LpsB, LpsC, LpsD, and LpsE glycosyl transferases are also homologous to the corresponding orthologues of *S. meliloti* 1021 and *S. fredii* NGR234, although the aminoacid identity is higher with NGR234 (92–96%) than with *S. meliloti* 1021 (80–83%).

At least two different *S. fredii* HH103 mutants in each of the *greA*, *lpsB* and *lpsE* genes were generated by using the *lacZ*Δp-Gm^R^ cassette or the omega (Ω) interposon. All the *S. fredii* HH103 *lpsB* (SVQ613 and SVQ615) and *lpsE* (SVQ642 and SVQ647) mutants showed altered LPS profiles, which consisted in an increase of the electrophoretic mobility of all bands composing the smooth (S-) and rough (R-) LPS (Figure 2A). The S-LPS of *lpsB* mutants migrated faster than those of the *lpsE* mutants. Thus, the altered LPS forms produced by *S. fredii* HH103 *lpsE* or *lpsB* mutants are structurally different. Inactivation of either *lpsB* or *lpsE* provoked that the LPS structures formed were no longer recognized by the monoclonal antibody NB6-228.22 (Figure 2B). These results are in line to those previously described for *S. meliloti* 1021 *lpsB* and *lpsE,* and *S. fredii* NGR234 *lpsB* mutants [9]–[11], [25]. We conclude that, similarly to these mutants, the core region of the LPS produced HH103 *lpsB* and *lpsE* mutants is altered.

*S. fredii* SVQ655 (*greA*::*lacZ*Δp-Gm^R^) does not show any clear alteration of its LPS electrophoretic profile, indicating that the *greA* gene is not directly involved in determining the LPS structure. We have also shown that *greA* and *lpsB* are not cotranscribed, which is in accordance to previous reports in *S. meliloti* 1021 [11]. However, our data (qPCR experiments) indicate that inactivation of *greA* has a negative effect on the expression level of *lpsB*. The decrease in *lpsB* expression is severe in SVQ656 but moderate in SVQ655. This difference might be attributed to the fact that, in SVQ655, *greA*, the *lacZ*Δp-Gm^R^ cassette, and *lpsB* are all transcribed in the same orientation and it is already known that the promoter of the Gm^R^ gene of the cassette (*aacC1*of *Tn*1696) directs transcription of genes located downstream [34]. In contrast, the transcription of *greA* and *lpsB* in strain SVQ656 is opposite to that of *aacC1*, so that the gentamycin-resistance cassette is not promoting the transcription of *lpsB*. Thus, we concluded that the inactivation of the *greA* gene in SVQ656 has an effect on the LPS structure because the absence of GreA negatively affects the transcription of *lpsB*. This effect would be masked in SVQ655 because the *aacC1* gene promotes transcription of downstream genes.

SVQ656 shows an LPS profile (Figure 2A, lane 11) that is different from those observed in *lpsB* (SVQ613 and SVQ615) and *lpsE* (SVQ642 and SVQ647) mutants. Hence, inactivation of *greA*, *lpsB*, and *lpE* causes different alterations on the LPS structure. As commented above, mutants SVQ655 and SVQ656 produce different patterns of silver-stained LPS bands (Figure 2A). These differences, however, are not observed in inmunostaining experiments using the monoclonal antibody (Figure 2B), suggesting that the altered LPS profile of the SVQ656 LPS might be originated by the generation of a complex mix of wild-type and truncated LPS forms, and that the latter forms have lost the epitope recognized by NB6-228.22. Both *S. fredii greA* mutants, but not those affected in either *lpsB* or *lpsE*, showed reduced swimming motility regardless whether LPS production was affected (SVQ656) or not (SVQ655), which indicates that this reduction in motility is not due to the reduced expression of *lpsB*. We have also shown in this work that *greA* does not affect the production of other surface polysaccharides, such as KPS, CG, and EPS, which in *S. fredii* HH103 have been related to bacterial motility [19], [23]. Further studies are required to elucidate the observed effect of *greA* on HH103 swimming ability. In fact, GreA is a transcription elongation factor that in *Escherichia coli* affects the expression of more than one hundred genes [35], [36]. SVQ656 was the only mutant tested showing an increase in the intensity of auto-agglutination. Paradoxically, neither the knock-out *lpsB* mutants (SVQ613 and SVQ615) nor SVQ655 (a *greA* mutant in which transcription of *lpsB* is about 17-fold higher than that of SVQ656) are affected in auto-agglutination. It might be possible that the altered LPS produced by SVQ656 (which is different to that produced by *lpsB* or *lpsE* mutants) caused disturbances in the bacterial surface that affect auto-agglutination.

Soybean plants inoculated with the *S. fredii* HH103 *lpsB* mutants (SVQ613 and SVQ615) formed many Fix^-^ pseudonodules (50–100 per root) and a few nodules of normal external appearance. Soybean plants inoculated with SVQ642 and SVQ647 (*lpsE* mutants) also gave similar responses, although the number of nodules formed by *lpsE* mutants was higher than that induced by mutants in *lpsB* (Table 2). In all cases, soybean plants showed clear symptoms of nitrogen starvation and their plant-top dry weight was not different from that of uninoculated plants. Soybean Williams roots inoculated with *S. fredii* HH103 *greA* mutants also formed pseudonodules and nodules. Although the number of nodules formed by the *greA* mutants was clearly higher than that induced by the *lpsB* mutants, plant-top dry weights were not different. Hence, regardless the number of nodules induced by the different mutants tested (Table 2), the aspect of the inoculated plants was very similar and clearly showed an absence of sustainable levels of nitrogen fixation (Figure S4). In contrast, *Vigna unguiculata* roots inoculated with mutant SVQ613 formed a considerable number of nitrogen fixing nodules (Table 2), indicating that the LPS alterations caused by the mutation in *lpsB* were not so deleterious for the symbiotic capacity of *S. fredii* HH103 with this legume.

Previously described *B. japonicum* and *B. elkanii* LPS mutants only induced the formation of pseudonodules on soybean roots, precluding the possibility to investigate any symbiotic role that LPS might play in mature soybean nodules [2], [13], [14]. In contrast, although *S. fredii* HH103 *greA*, *lpsB*, and *lpsE* mutants also induce the formation of numerous pseudonodules, they are still able to induce the formation of some soybean nodules of normal external morphology. Hence, the importance of LPS in fully developed soybean nodules can be studied in the *S. fredii* HH103-soybean symbiosis. Pseudonodules induced by mutants SVQ656 (*greA*) and SVQ613 (*lpsB*) contained the vascular bundles in a central position (Figure S6) instead of in the periphery as it occurs in normal soybean nodules (Figure 5A), indicating that nodule morphogenesis has been disturbed at early stages. Thus, apart other possible functions, LPS might be acting as a symbiotic signal in early stages of the nodule developmental program in the *S. fredii* HH103-soybean symbiosis. The structure of soybean nodules of normal external morphology induced by *S. fredii* HH103 Rif^R^ or its mutant derivatives SVQ656 (*greA*), SVQ613 (*lpsB*), and SVQ642 (*lpsE*) were compared by light and transmission electron microscopy. Infection thread formation, bacterial release into the host cells, and nodule zonation were similar in all cases. However, some aspects of symbiosome development and cell wall structure of nodule cells infected by any of the mutants showed clear alterations in respect to those occupied by the wild type strain. These alterations are: i) symbiosomes are maintained as single-bacteroid units instead of normal multi-bacteroid symbiosomes, an indication of symbiosome immaturity and possible defects in bacteroids division inside of symbiosomes and/or homotypic fusion of symbiosomes; ii) early termination of symbiosis indicated by huge peribacteroid spaces, numerous vesicles fusing with peribacteroid membrane and appearance of “ghost membranes”, which are clear indications of bacteroid lysis; iii) the presence of abnormal osmiofilic and electron-dense deposits; and iv) the presence of thick cell walls in infected cells, which are particularly visible in 35 day-old nodules induced by SVQ656 and indicate the occurrence of plant defense reactions. To our knowledge, this is the first study that describes an effect of a rhizobial LPS mutant on plant cell wall structure of an infected cell.

Our results indicate that *S. fredii* HH103 *greA*, *lpsB*, and *lpsE* mutants can induce the formation of nodule primordia on soybean roots. Very frequently, however, these primordia do not end in the formation of truly structured nodules but in aborted pseudonodules devoid of bacteria. All the mutants tested could also induce the formation of some nodules of normal external morphology, although the aspect of the soybean plants indicated that nitrogen fixation was too low to meet their demand of nitrogen. TEM images indicated that fully-developed nodules induced by the *S. fredii* HH103 *greA*, *lpsB* or *lpsE* mutants are not able to sustain stable symbiotic interactions since nodule senescence is initiated soon after the symbiosis becomes functional or even before of symbiosome maturation. The elimination of the microsymbiont by lytical clearance before nitrogen-fixing multi-bacteroid symbiosomes are formed, together with the presence of osmiophilic and electron-dense deposits, could be a sign of soybean defense reactions.

In summary, all these results indicate that in the *S. fredii* HH103-soybean symbiosis the bacterial LPS would play symbiotic role(s) at early and late stages of the nodulation process. *S. fredii* HH103 LPS would also contribute to symbiosome stability in this determinate-nodule forming symbiosis. Similarly, the *S. meliloti lpsB* gene is also necessary for the maintenance of a chronic intracellular infection in alfalfa nodules, an indeterminate-nodule forming legume [33]. Thus, regardless the type of nodule formed the rhizobial LPS appears necessary for the stability of the intracellular symbiosis and resembles the situation described for some *Brucella abortus* LPS mutants, which exhibits decreased survival in macrophages and greatly accelerated clearance from experimentally infected mice compared to the virulent parental strain [37].

## Materials and Methods

### Molecular and Microbiological Techniques

The bacterial strains and plasmids used in this work are listed in Table 1. *Sinorhizobium fredii* strains were grown at 28°C on TY medium [38], yeast extract/mannitol (YM) medium [39] or MGM medium [40]. *Escherichia coli* was cultured on Luria-Bertani (LB) medium [41] at 37°C. When required, the media were supplemented with the appropriate antibiotics as described by Vinardell et al. [31]. Flavonoids were dissolved in ethanol at a concentration of 1 mg ml^−1^ and used at 1 µg ml^−1^. Plasmids were transferred from *E. coli* to rhizobia by triparental conjugation as described by Simon [42] and using pRK2013 as the helper plasmid. The Optical Density (O.D.) of bacterial cultures or enzymatic reactions was determined by using a Pharmacia LKB Novaspec II spectrophotometer.

Assays to determine the sensitivity of *S. fredii* HH103 and its mutant derivatives to different toxic or stressing chemical compounds were carried out as follows: Petri plates containing solid TY media were overlaid with 4 ml of soft water-agar [0.7% (W/V) agar in distilled water] carrying 10^5^ bacteria. Paper disks (Whatman, 6 mm of diameter) impregnated with 5 µl of a stock solution containing the compound to be tested were placed on top of the water-agar layer. Then, the occurrence of bacterial growth inhibition around the disks (assessed by the presence of inhibition halos measured from the centre of the disk) was scored at the third day of incubation. The chemicals tested and the stock solutions used were as follows: vanillic acid, ferulic acid or salicylic acid (stock solutions at 25, 50, and 100 mM in ethanol), sodium dodecyl sulphate (SDS, stock solution at 6.9 mM in water), ethanol (100% V/V), genistein (stock solution at 3.7 mM in ethanol), hydrogen peroxide (stock solutions at 25, 50, and 100 mM V/V), paraquat (also called methyl viologen dichloride, stock solution at 1 M in water).

Recombinant DNA techniques were performed according to the general protocols of Sambrook et al [41]. For hybridization, DNA was blotted to Amersham Hybond™-N nylon membranes, and the DigDNA method of Roche was employed according to manufacturer’s instructions. PCR amplifications were performed as previously described [43]. Primers were designed by using the GeneFisher2 utility. The National Center for Biotechnology Information (NCBI) BLASTN and BLASTP programs were used for homology searches (against databases “nucleotide collection (nr/nt)” and “non-redundant protein sequences (nr)”, respectively). All primer pairs used in this work are listed in Table S1. DNA fragments were visualized by agarose (2.0 or 0.8%, W/V) gel electrophoresis and ethidium bromide staining.

### Construction of Mutants

In order to generate *lps* and *greA* mutants of *S. fredii* HH103, we constructed derivatives of pK18mob [44], a suicide vector in rhizobia, carrying an internal fragment of the corresponding gene interrupted by the *lacZ*Δp-Gm^R^ cassette [45] or the Omega interposon [46]. In the case of *lpsB* mutants, Ω and *lacZ*Δp-Gm^R^ were subcloned as 2-kb and 4.5-kb *Sma*I fragments into the unique *Nru*I site present in the gene. To mutate *lpsE* and *greA*, the *lacZ*Δp-Gm^R^ was subcloned into these genes as a 4.5-kb *Bam*HI or a 4.5-kb *Sal*I fragment respectively. In the latter gene, digestion with *Sal*I removes a 279-bp internal fragment. The plasmids generated (see Table 1), named pMUS982 (*lpsB*::*lacZ*Δp-Gm^R^→→), pMUS990 (*lpsB*:: Ω), pMUS1008 (*lpsE*::*lacZ*Δp-Gm^R^→→), pMUS1020 (*lpsE*::*lacZ*Δp-Gm^R^→←), pMUS1022 (*greA*::*lacZ*Δp-Gm^R^→←), and pMUS1023 (*greA*::*lacZ*Δp-Gm^R^→→) were individually transferred to HH103-Rif^R^, and Rif^R^ Spc^R^ Km^S^ (when the Ω interposon was used) or Rif^R^ Gm^R^ Km^S^ (when the *lacZ*Δp-Gm^R^ cassette was employed) transconjugants were identified in order to isolate double recombinants in which the wild-type *lps* or *greA* gene had been substituted by the mutated copy of the gene. In all cases, homogenotization of the mutated version of the *lps* or *greA* gene was confirmed by PCR and DNA-DNA hybridization.

### Complementation Studies

Cosmid pMUS908 carries the *S. fredii* HH103 *greA*, *lpsB*, *lpsE*, *lpsD*, and *lpsC* genes. *S. fredii* HH103 *greA* mutants carrying cosmid pMUS908 regained the wild-type phenotype concerning LPS electrophoretic profile, recognition by the monoclonal antibody NB6-228.22, bacterial mobility, and symbiotic capacity with soybean cv. Williams. However, none of the defects exhibited by *lpsB* and *lpsE* mutants were complemented *in trans* by cosmid pMUS908.

Attempts to complement *S. fredii* HH103 *lps* mutants were carried out by transferring genes cloned in plasmids that are stable (*in trans* complementation) or suicide (*in cis* complementation) in this bacterial strain. For *in cis* complementation of *S. fredii lpsE* mutants (SVQ642 and SVQ647), a 2.5-kb *Eco*RI-*Sph*I fragment of cosmid pMUS908 carrying the 3′-end of *lpsB*, the complete *lpsE* and the 3′-end of *lpsD* was subcloned into pK18mob, generating plasmid pMUS1005. This plasmid was transferred to strains SVQ642 and SVQ647 and Rif^R^ Gm^R^ Km^R^ clones were selected in order to obtain putative single recombinants in which the wild type *lpsE* gene had been integrated in the genome next to the mutated copy of the gene. In these candidates, the presence of a wild-type copy of the *lpsE* gene was checked by PCR using primers *lpsE*-F and *lpsE*-R (Table S1) which lead to the amplification of a *lpsE* 481-bp internal fragment which contains the *Bam*HI site in which the *lacZ*Δp-Gm^R^ cassette was inserted to generate SVQ642 and SVQ647 mutants. The resultant strains, SVQ642C1 and SVQ647C2, regained the wild-type phenotype concerning LPS electrophoretic profile, recognition by the monoclonal antibody NB6-228.22, and symbiotic capacity with soybean cv. Williams.

Several attempts have been carrying out to complement *S. fredii* HH103 *lpsB* mutants. First, a 1.4-kb fragment containing a wild type copy of the *lpsB* gene including its promoter region was amplified by PCR using *greAlpsB*-F and *lpsBE*-R primers (Table S1) and subcloned into the broad-host-range plasmid pCPP46, generating plasmid pMUS1139. This plasmid was transferred to strain SVQ613 and Rif^R^ Spc^R^ Tc^R^ transconjugants were selected. In these candidates, the presence of a wild-type copy of the *lpsB* gene was checked by PCR using primers *greAlpsB*-F and *lpsBE*-R. These candidates showed two bands: one (1.4-kb) corresponding to the wild-type *lpsB* gene and the other (3.4-kb) to the mutated *lpsB* copy. In order to complement *in cis* the *lpsB* mutants, the 1.4-kb fragment containing the wild type copy of the *lpsB* gene was subcloned into the pK18mob vector to generate plasmid pMUS1144. This plasmid was transferred to strain SVQ613 and Rif^R^ Spc^R^ Km^R^ transconjugants were analysed by PCR in order to test if they had a reconstituted wild-type copy of the *lpsB* gene. For unknown reasons, none of these strategies led to an effective complementation of *lpsB* mutants.

### Motility, Auto-agglutination and Biofilm Formation Assays

Motility assays were performed as previously described [23]. For bacterial auto-agglutination assays, liquid YM cultures of *S. fredii* strains were incubated at 28°C on an orbital shaker at 180 rpm until they reached the early-stationary phase (about O.D._660_ = 0.8). Then, 4.5 ml aliquots of bacterial cultures were transferred to tubes (generating liquid columns of 4 cm high) and incubated at 28°C under static conditions for 68 hours. In order to estimate bacterial auto-agglutination, YMB liquid cultures of *S. fredii* strains at their early stationary-growth phase (optical density at 600 nm [OD600] >0.8) were left in static at room temperature; auto-agglutination was estimated by time-course measures of the O.D._660_ of the cultures at a deepness of 2.5 cm from the liquid-air interphase.

Biofilm formation on plastic surfaces was studied by using the method of O’Toole and Kolter [47], with some modifications. Cultures were grown in low-phosphate MGM medium as described above, diluted to an optical density at 600 nm (OD_600_) of 0.2, and inoculated into microtiter polystyrene plate wells in 100-µl aliquots. The plates were covered with a sterile lid to prevent evaporation, turned upside down, and incubated without agitation at 28°C for 48 h. Bacterial growth was quantified by measuring the OD_600_ at the end of the experiment. The contents of each well were then removed, and the wells were dried at room temperature and washed three times with 100 µl of sterile physiological saline solution in order to remove all non-adherent bacteria. The plates were emptied, air dried, stained for 20 min with 100 µl of 0.1% crystal violet per well, air dried, then rinsed three times with water and air dried. Biofilm formation was quantified by the addition of 100 µl of 96% ethanol to each crystal violet-stained microtiter dish well, and the absorbance (at a wavelength of 570 nm) of the solubilized crystal violet was determined with a microplate reader (Synergy HT; BioTek; Winooski, Vermont, USA). Bacterial growth and adherence measurements were performed in duplicate. Data presented are the media of at least three independent experiments performed in duplicate; in each experiment, at least 12 wells per treatment were measured.

### Gene Expression Analysss

Assays for β-galactosidase activity in liquid bacterial cultures on YMB were carried out 16 h after induction as previously described [22], [31]. At least three independent experiments performed in duplicate were carried out.

For RT-PCR analysis, *S. fredii* strains were incubated in YMB in an orbital shaker (180 rpm) at 28°C. When the cultures reached an OD_660_ of 0.3–0.4, cells were harvested, and RNA was extracted by using the RNAprotect Bacteria Reagent, the RNAeasy mini kit, and the RNase-free DNase Set (all provided by Qiagen, Basel, Switzerland) following the manufacturer’s instructions. To ensure that genomic DNA was not present in RNA samples, a control PCR was done employing primers *lpsE*-F and *lpsE*-R (Table S1). Only those RNA samples showing no PCR amplification were selected for further work. Reverse transcription of total RNA was carried out using the Quantitect kit (Qiagen), which includes a genomic DNA elimination step before RNA reverse transcription. RT-PCR products were visualized by agarose gel electrophoresis and ethidium bromide staining.

*q*PCR experiments were performed in a 20-µl final volume containing 1 µl of cDNA, 0.6 pmol of each primer, and 10 µl of FastStart SYBR Green Master Mix (Roche Diagnostics). PCR was conducted on the iCycler IQ (Bio-Rad Laboratories SA, Marnes La Coquette, France), and the threshold cycles were determined with the iCycler software. Primers used for amplification of a 135-bp internal fragment of the *S. fredii* HH103 *lpsB* cDNA were q*lpsB*-F and q*lpsB*-R (Table S1). To normalize the data, a 197-bp internal fragment of the *S. fredii* HH103 16 S rRNA (accession number AY260145) was employed as an internal control in each sample by using primers HH16S-F and HH16S-R (Table S1). For each strain the relative expression of *lpsB* with regard to that of HH103 was calculated by using the formula 2^ΔΔCT^, where **ΔΔ**C_T_ = (C_T *lpsB*_ - C_T 16S_) problem strain - (C_T *lpsB*_- C_T 16S_) HH103. Three independent experiments performed in triplicate were carried out and averaged.

### Analysis of Lipopolysaccharide (LPS) and K-antigens (KPS)

LPS extraction from bacterial cultures grown on solid TY medium, separation on SDS-PAGE gels and silver staining were performed as described previously [48]. Immuno-staining procedures and the monoclonal antibody NB6-228.22 were as described by Buendía-Clavería et al. [48]. K-antigen capsular polysaccharides (KPS) were extracted from bacterial cultures grown on solid TY medium and analysed by PAGE as described by Hidalgo et al. [21].

The detection of *S. fredii* KPS and cyclic glucans (CG) by Nuclear Magnetic Resonance (NMR) was performed as previously described [19], [21].

### Nodulation Tests

Nodulation assays on *Glycine max* (L.) Merr. cv. Williams and *Vigna unguiculata* (L.) cv. Bisbee Red were carried out as previously described [49]. Germinated seeds were transferred to Leonard jars containing sterilized vermiculite supplemented with Fähraeus nutrient solution [39]. Each plant was inoculated with approximately 10^8^ bacteria and then grown for at least 6 weeks with a 16-h photoperiod at 25°C in the light and 18°C in the dark. Plant tops were dried at 70°C for 48 h and weighed. Bacterial isolation from surface-sterilized nodules was carried out as previously described [49].

### Light and Transmission Electron Microscopy

Soybean nodules from 3- and 5-week old plants were collected and immediately fixed in a mix of 5% glutaraldehyde, 4% paraformaldehyde in Na-cacodylate buffer, were further postfixed in 1% osmium tetroxide, dehydrated in an ethanol series and progressively embedded in LR White resin (London Resin Co., Reading, UK) as described by Shvaleva et al. [50].

Semithin (1 µm) and ultrathin (70–90 nm) sections were cut with a Reichert Ultracut S ultramicrotome (Leica, Vienna, Austria) fitted with a diamond knife. Semithin sections for light microscopy were stained with 1% (w/v) toluidine blue in aqueous 1% sodium borate for direct observation with a Zeiss Axiophot photomicroscope (Oberkochen, Germany) and photographed using a Leica digital camera. Ultrathin sections were contrasted with a solution with 2% of uranil acetate and Reinolds lead citrate and examined using a STEM LEO 910 electron microscope (Oberkochen, Germany) at accelerating voltage of 80 kV, equipped with a Gatan Bioscan 792 digital camera (Pleasanton, CA, USA). Different sections from at least four different nodules were analyzed.

## Supporting Information

Figure S1 **Polyacrylamide gel electrophoresis analysis of K-antigen polysaccharide (KPS) production by** ***Sinorhizobium fredii*** **HH103 Rif^R^ and its** ***lpsB*** **(SVQ613),** ***lpsE*** **(SVQ642) and** ***greA*** **(SVQ655 and SVQ656) mutant derivatives.** Samples were run in the absence of detergent (SDS), treated with Alcian Blue, and silver stained. Lanes 1, 3, and 7, HH103 Rif^R^; lane 2, SVQ613; lane 4 SVQ642; lane 5, SVQ655; and lane 6, SVQ656.
(TIF)

Figure S2 **Nuclear magnetic resonance (NMR) analysis of K-antigen polysaccharide (KPS) and cyclic glucans (CG) production by** ***Sinorhizobium fredii*** **strains.** ^1^H-NMR spectra (500 MHz) of crude cell extracts containing surface polysaccharides isolated from *S. fredii* HH103 Rif^R^, SVQ613 (*lpsB*::Ω), SVQ656 (*greA*::*lacZ*Δp-Gm^R^), and SVQ642 (*lpsE*::*lacZ*Δp-Gm^R^). Signals corresponding to KPS, cyclic glucans (CG), or the solvent (HDO, deuterated water) are indicated.
(TIF)

Figure S3 ***S. fredii*** **HH103** ***greA*** **,** ***lpsB*** **or** ***lpsE*** **mutants are not significantly affected in their capacity to form biofilms.** Bacterial biofilm formation was estimated by the relation OD_570_/OD_600_ obtained for the different bacterial cultures.
(TIF)

Figure S4 **Plant responses to inoculation of** ***Glycine max*** **cv. Williams with** ***Sinorhizobium fredii*** **HH103 Rif^R^ and different** ***greA*** **,** ***lpsB*** **, and** ***lpsE*** **mutant derivatives.** Aerial parts of soybean plants inoculated with: A, HH103 Rif^R^; B, SVQ613 (*lpsB*::Ω); C, SVQ615 (*lpsB::lacZ*Δp-Gm^R^); D, uninoculated control; E, SVQ642 (*lpsE*::*lacZ*Δp-Gm^R^); F, SVQ642 carrying cosmid pMUS908; G, SVQ647 (*lpsE*::*lacZ*Δp-Gm^R^); H, SVQ647 pMUS908; I, SVQ655 (*greA*::*lacZ*Δp-Gm^R^); J, SVQ655 pMUS908; K, SVQ656 (*greA*::*lacZ*Δp-Gm^R^); and L, SVQ656 carrying pMUS908.
(TIF)

Figure S5 **Light microscopy images of pseudonodules elicited in soybean roots by SVQ656 (g** ***reA*** **) and SVQ613 (** ***lpsB*** **).** Vascular tissues (V) in a central zone are surrounded by sclereid cells (arrow). Bar size: 500 µm.
(TIF)

Figure S6 **Light microscopy images of 21-dpi nodules elicited by** ***Sinorhizobium fredii*** **strains HH103 Rif^R^ (A–C) and SVQ656 (g** ***reA*** **) (D–F).** A, D, general view of a nodule showing cortex zone (c), meristem and infection zone (i) and infected zone (iz). B, E, detail of infection zone, freshly infected cells and more mature infected cells. C, F, detail of mature infected cells. Note that differences between nodules induced by HH103 and SVQ656 are only observed in mature infected cells but not in freshly infected cells.Bar size: A, D: 500 µm; B, E: 10 µm; C, F: 20 µm.
(TIF)

Table S1 **Primers used in PCR experiments.**
(DOC)
