# Supplementary figures and images for: The Sinorhizobium fredii HH103 Lipopolysaccharide Is Not Only Relevant at Early Soybean Nodulation Stages but Also for Symbiosome Stability in Mature Nodules
Source: PLoS One. 2013 Oct 1;8(10):e74717. doi: 10.1371/journal.pone.0074717 (PMC3788101; doi:10.1371/journal.pone.0074717)

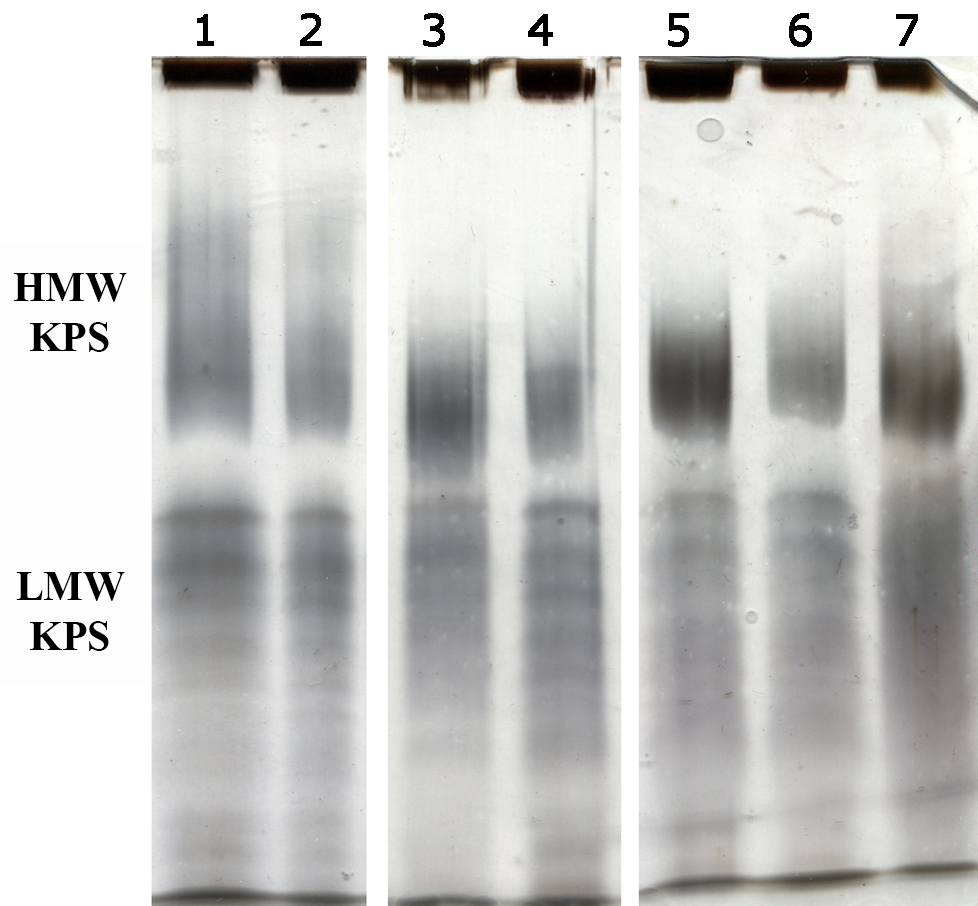

Supplement: Figure S1 — Polyacrylamide gel electrophoresis analysis of K-antigen polysaccharide (KPS) production by Sinorhizobium fredii HH103 RifR and its lpsB (SVQ613), lpsE (SVQ642) and greA (SVQ655 and SVQ656) mutant derivatives. Samples were run in the absence of detergent (SDS), treated with Alcian Blue, and silver stained. Lanes 1, 3, and 7, HH103 RifR; lane 2, SVQ613; lane 4 SVQ642; lane 5, SVQ655; and lane 6, SVQ656. (TIF) [file pone.0074717.s001.tif]

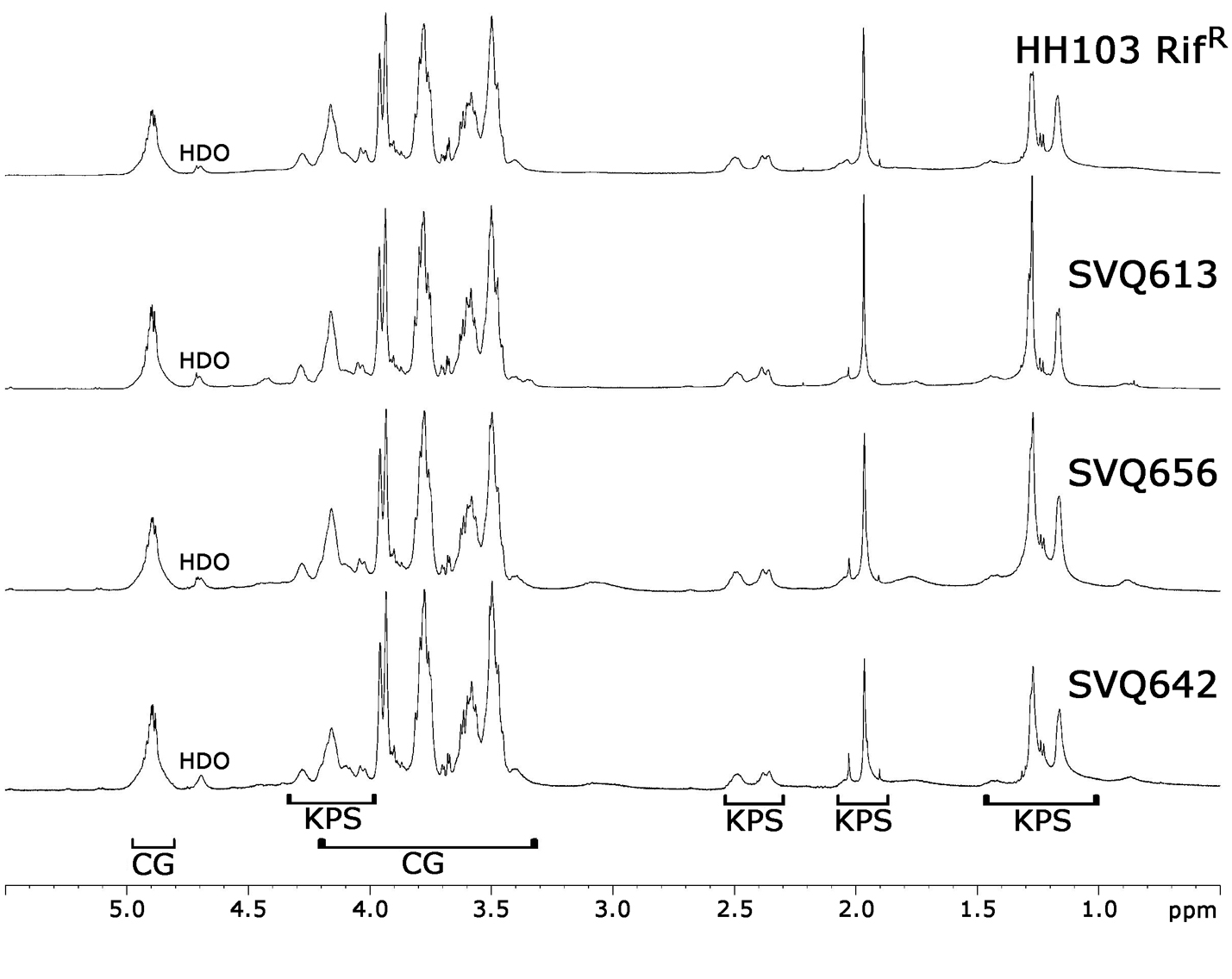

Supplement: Figure S2 — Nuclear magnetic resonance (NMR) analysis of K-antigen polysaccharide (KPS) and cyclic glucans (CG) production by Sinorhizobium fredii strains. 1H-NMR spectra (500 MHz) of crude cell extracts containing surface polysaccharides isolated from S. fredii HH103 RifR, SVQ613 (lpsB::Ω), SVQ656 (greA::lacZΔp-GmR), and SVQ642 (lpsE::lacZΔp-GmR). Signals corresponding to KPS, cyclic glucans (CG), or the solvent (HDO, deuterated water) are indicated. (TIF) [file pone.0074717.s002.tif]

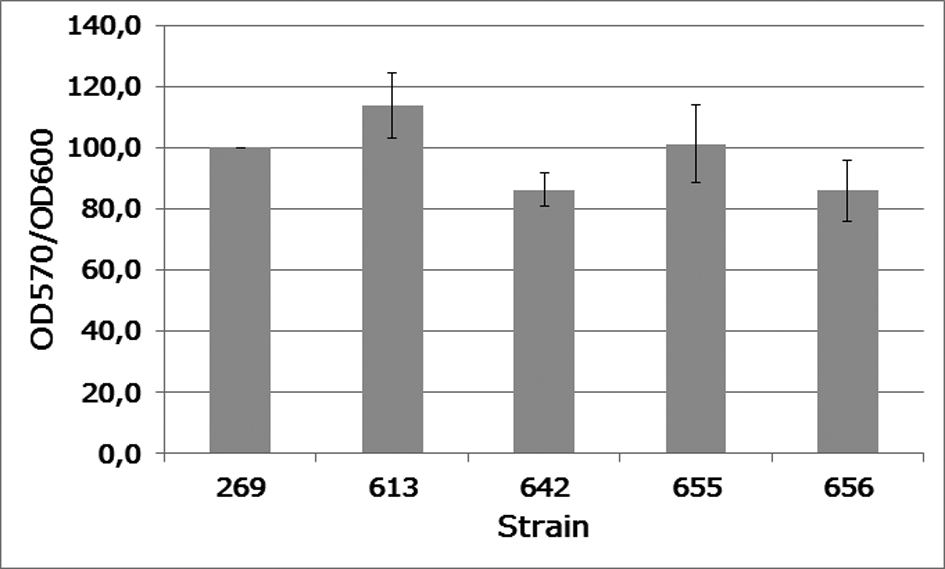

Supplement: Figure S3 — S. fredii HH103 greA , lpsB or lpsE mutants are not significantly affected in their capacity to form biofilms. Bacterial biofilm formation was estimated by the relation OD570/OD600 obtained for the different bacterial cultures. (TIF) [file pone.0074717.s003.tif]

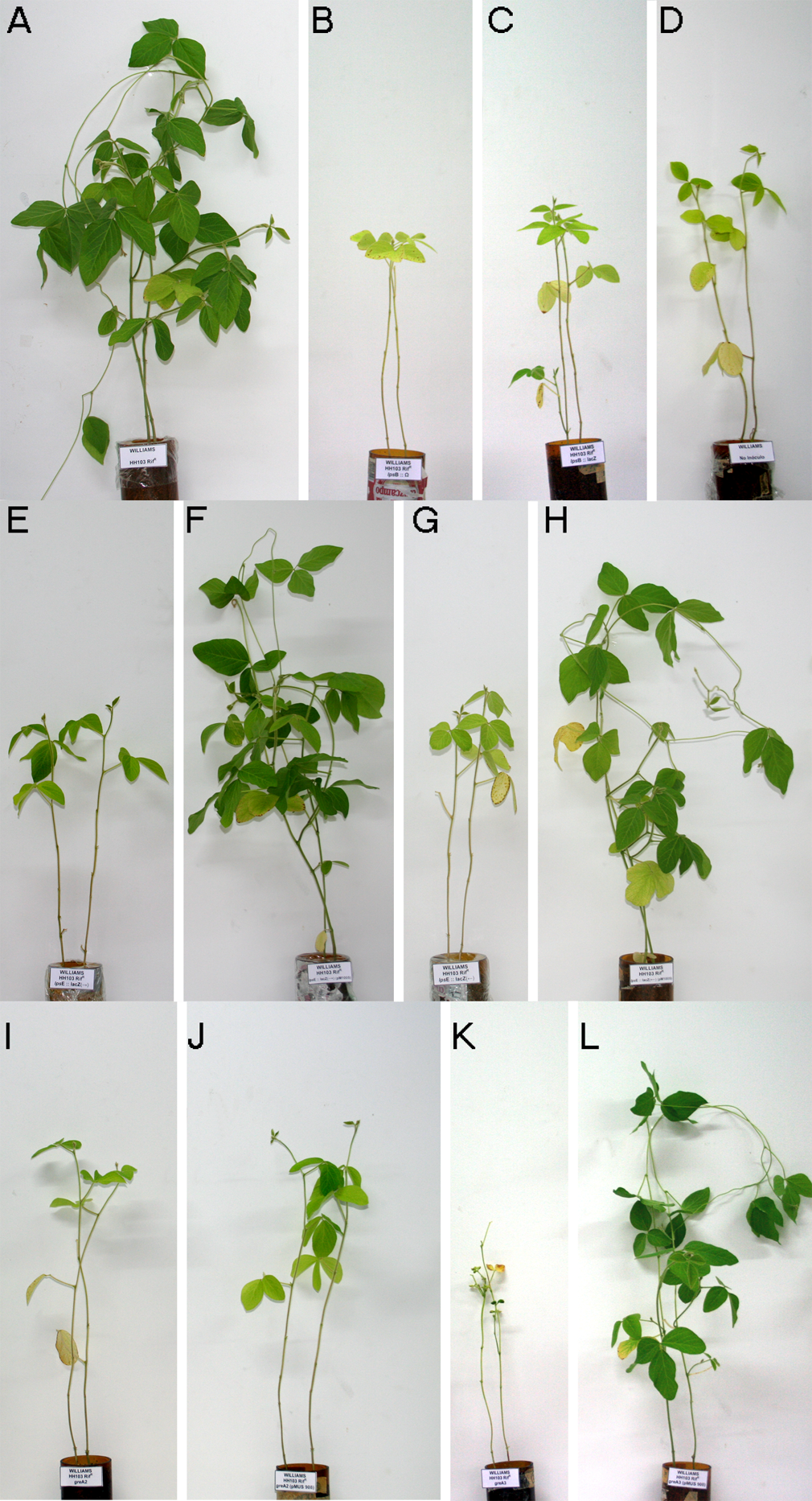

Supplement: Figure S4 — Plant responses to inoculation of Glycine max cv. Williams with Sinorhizobium fredii HH103 RifR and different greA , lpsB , and lpsE mutant derivatives. Aerial parts of soybean plants inoculated with: A, HH103 RifR; B, SVQ613 (lpsB::Ω); C, SVQ615 (lpsB::lacZΔp-GmR); D, uninoculated control; E, SVQ642 (lpsE::lacZΔp-GmR); F, SVQ642 carrying cosmid pMUS908; G, SVQ647 (lpsE::lacZΔp-GmR); H, SVQ647 pMUS908; I, SVQ655 (greA::lacZΔp-GmR); J, SVQ655 pMUS908; K, SVQ656 (greA::lacZΔp-GmR); and L, SVQ656 carrying pMUS908. (TIF) [file pone.0074717.s004.tif]

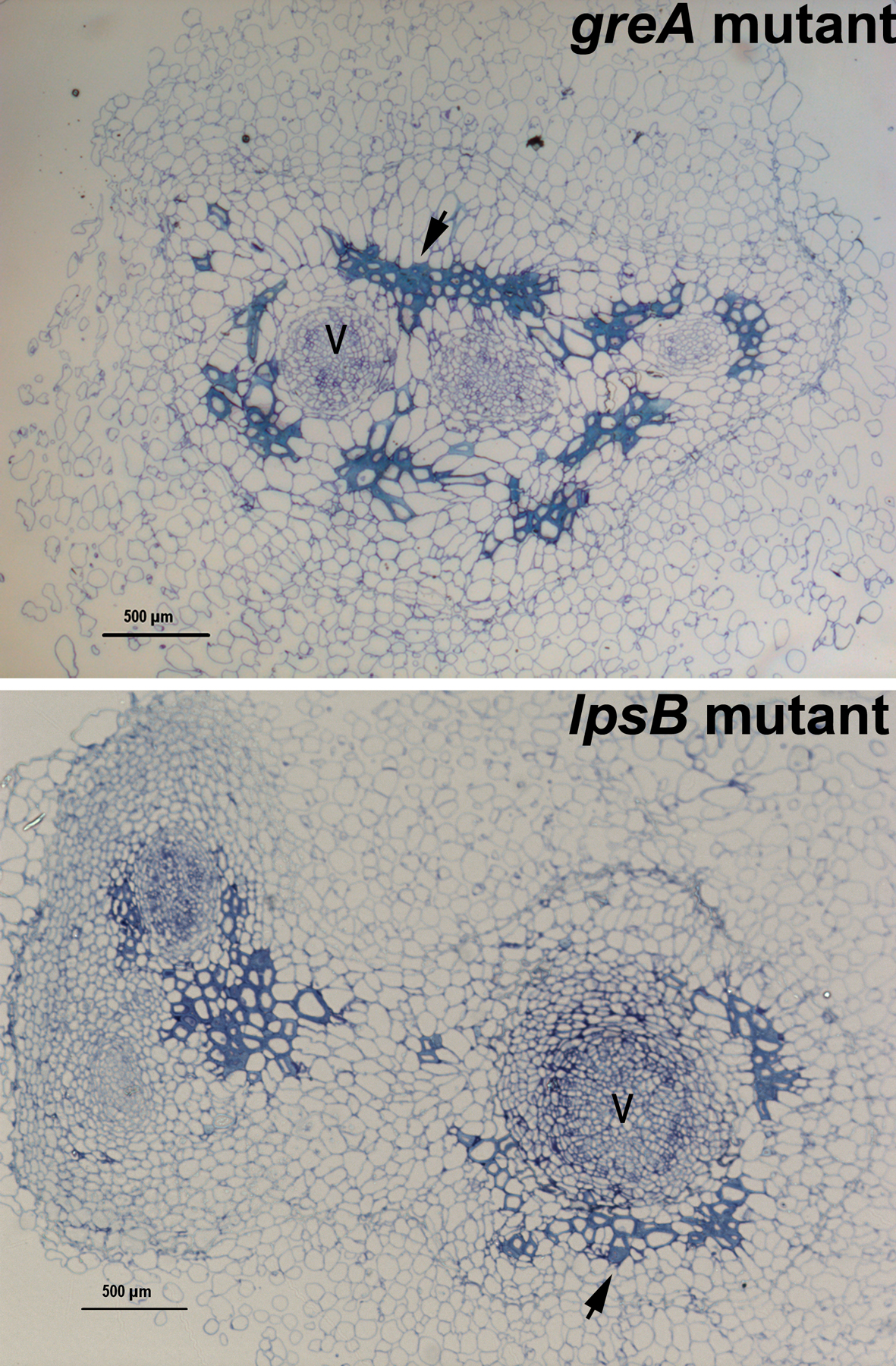

Supplement: Figure S5 — Light microscopy images of pseudonodules elicited in soybean roots by SVQ656 (g reA ) and SVQ613 ( lpsB ). Vascular tissues (V) in a central zone are surrounded by sclereid cells (arrow). Bar size: 500 µm. (TIF) [file pone.0074717.s005.tif]

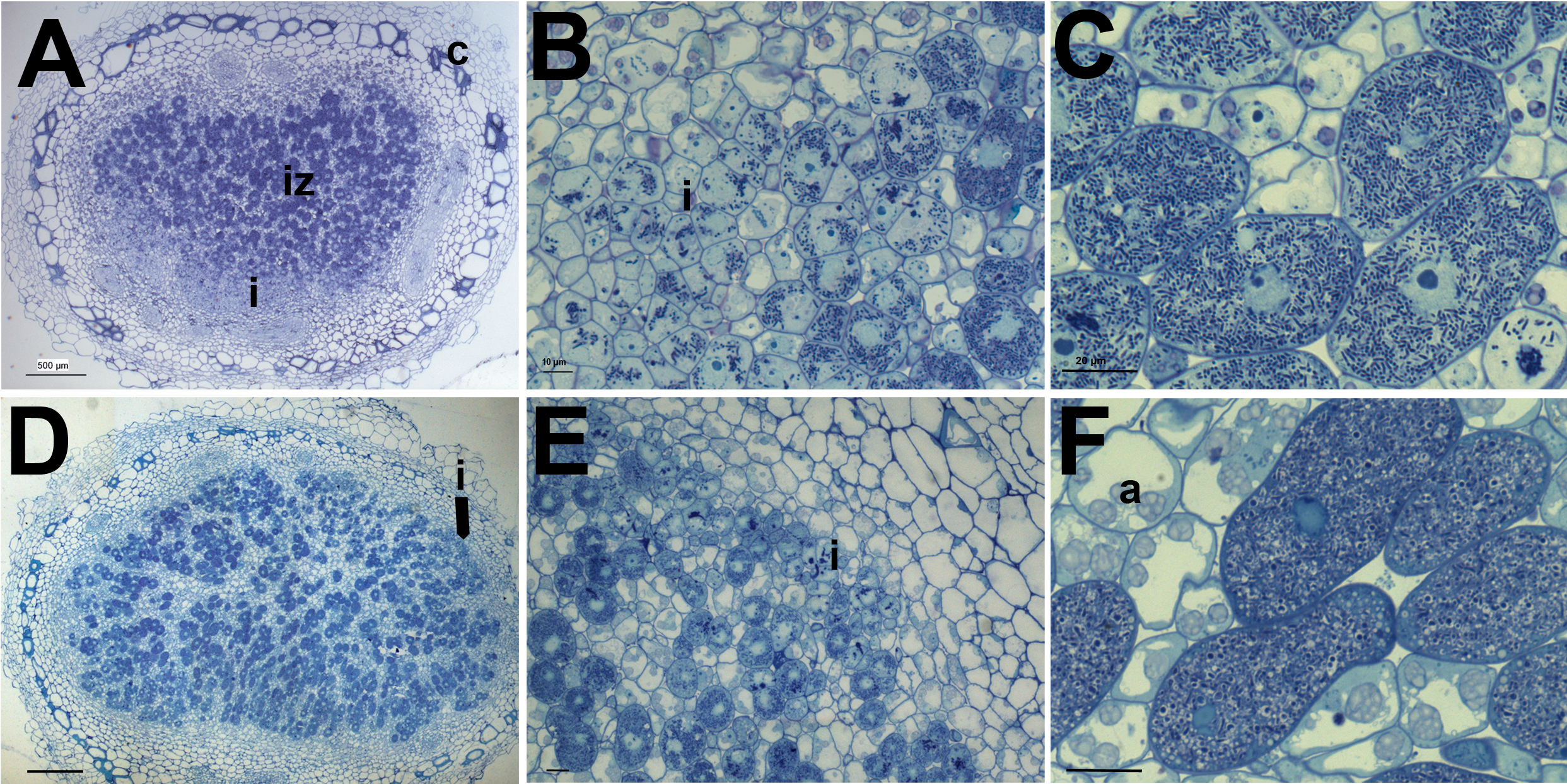

Supplement: Figure S6 — Light microscopy images of 21-dpi nodules elicited by Sinorhizobium fredii strains HH103 RifR (A–C) and SVQ656 (g reA ) (D–F). A, D, general view of a nodule showing cortex zone (c), meristem and infection zone (i) and infected zone (iz). B, E, detail of infection zone, freshly infected cells and more mature infected cells. C, F, detail of mature infected cells. Note that differences between nodules induced by HH103 and SVQ656 are only observed in mature infected cells but not in freshly infected cells.Bar size: A, D: 500 µm; B, E: 10 µm; C, F: 20 µm. (TIF) [file pone.0074717.s006.tif]
