# Supplementary material for: The Sinorhizobium fredii HH103 Lipopolysaccharide Is Not Only Relevant at Early Soybean Nodulation Stages but Also for Symbiosome Stability in Mature Nodules
Source: PLoS One. 2013 Oct 1;8(10):e74717. doi: 10.1371/journal.pone.0074717 (PMC3788101; doi:10.1371/journal.pone.0074717)
Supplement: Table S1 — Primers used in PCR experiments. (DOC) [file pone.0074717.s007.doc]

**Table S1.** Primers used in PCR experiments.

| **Primer** | **Sequence (5´-3´)** | **Priming site** | **Predicted length (bp)** | **Use** |
| --- | --- | --- | --- | --- |
| HH*lpsB-*F | 5´ccgggtggagcaagttcctga | *lpsB* internal | 418 | Genomic library screening |
| HH*lpsB-*R | 5´agggcgcggtaccaatcagga |  |  |  |
| *greAlpsB*-F | 5´gaggttggcgactcgatcga | 3’ end of *greA* | 1412 | *lpsB* amplification |
| *lpsBE*-R | 5´gcgatcgagcgaatcgccaa | 3’ end of *lpsE* |  |  |
| *lpsE*-F | 5’ cgcctcgactattttcgcaa | *lpsE* internal | 481 | wt *lpsE* screening |
| *lpsE*-R | 5’ cttcggagcgacttgagaca |  |  |  |
| *greA*int-F | 5’cgaataccatgccgccaagga | *greA* internal | 284 | RT-PCR experiments |
| *greA*int-R | 5’tcacctcgatcgagtcgccaa |  |  |  |
| *greAlpsB* F | 5’gaggttggcgactcgatcga | 3’ end of *greA* | 296 | RT-PCR experiments |
| *greAlpsB* R | 5’gctggatgatcgtcgaggtga | 5’ end of *lpsB* |  |  |
| rt*lpsB* F | 5’tcttcacctctgcctcgcaga | *lpsB* internal | 156 | RT-PCR experiments |
| rt*lpsB* R | 5’tcgatacgcatcggttccgtc |  |  |  |
| *lpsBE*-F | 5’ggtgcggccacattcatgga | 3’ end of *lpsB* | 282 | RT-PCR experiments |
| *lpsBE*-R | 5’gcgatcgagcgaatcgccaa | 3’ end of *lpsE* |  |  |
| *qlpsB*-F | 5´ ggtgtcacctcgacgatc | *lpsB* internal | 135 | *q*PCR experiments |
| *qlpsB*-R | 5´ cagaggtggatcaggtcg |  |  |  |
| HH16S-F | 5´ ggatcggagacaggtgctgca | 16S rRNA internal | 197 | *q*PCR experiments |
| HH16S-R | 5´ cgtgtgtagcccagcccgta |  |  |  |
